# Relationship between resting 12-lead electrocardiogram and all-cause death in patients without structural heart disease: Shinken Database analysis

**DOI:** 10.1186/s12872-021-01864-3

**Published:** 2021-02-10

**Authors:** Naomi Hirota, Shinya Suzuki, Takuto Arita, Naoharu Yagi, Takayuki Otsuka, Mikio Kishi, Hiroaki Semba, Hiroto Kano, Shunsuke Matsuno, Yuko Kato, Tokuhisa Uejima, Yuji Oikawa, Minoru Matsuhama, Mitsuru Iida, Tatsuya Inoue, Junji Yajima, Takeshi Yamashita

**Affiliations:** 1 Department of Cardiovascular Medicine, The Cardiovascular Institute, 3-2-19 Nishiazabu, Minato-Ku, Tokyo, 106-0031 Japan; 2 Department of Cardiovascular Surgery, The Cardiovascular Institute, Tokyo, Japan

**Keywords:** Electrocardiogram, Death, Mortality, Prediction

## Abstract

**Background:**

Resting 12-lead electrocardiography is widely used for the detection of cardiac diseases. Electrocardiogram readings have been reported to be affected by aging and, therefore, can predict patient mortality.

**Methods:**

A total of 12,837 patients without structural heart disease who underwent electrocardiography at baseline were identified in the Shinken Database among those registered between 2010 and 2017 (*n* = 19,170). Using 438 electrocardiography parameters, predictive models for all-cause death and cardiovascular (CV) death were developed by a support vector machine (SVM) algorithm.

**Results:**

During the observation period of 320.4 days, 55 all-cause deaths and 23 CV deaths were observed. In the SVM prediction model, the mean c-statistics of 10 cross-validation models with training and testing datasets were 0.881 ± 0.027 and 0.927 ± 0.101, respectively, for all-cause death and 0.862 ± 0.029 and 0.897 ± 0.069, respectively for CV death. For both all-cause and CV death, high values of permutation importance in the ECG parameters were concentrated in the QRS complex and ST-T segment.

**Conclusions:**

Parameters acquired from 12-lead resting electrocardiography could be applied to predict the all-cause and CV deaths of patients without structural heart disease. The ECG parameters that greatly contributed to the prediction were concentrated in the QRS complex and ST-T segment.

## Background

Prediction of the risk of all-cause death has been the cornerstone of epidemiology and allows for the readjustment of the current medical treatment and the modification of established risk factors (i.e., smoking cessation, statin therapy, or blood pressure control for cardiovascular disease) [1]. Predictive models for all-cause death have mostly been developed through the combination and weighting of patient characteristics, i.e., age, sex, and various comorbidities. However, because the risk of all-cause death is strongly affected by the stage of each disease, applying a simple, dichotomized category of disease (i.e., existence or absence) to the risk models would under- or overestimate the risk of all-cause death, as it ignores the wide range of individual differences in the disease status.

Resting 12-lead electrocardiography, which is a non-invasive and readily available test, is widely performed for the detection and management of cardiac diseases. Electrocardiography enables the evaluation of the risk of cardiac diseases in medical examinations. Many studies have shown that electrocardiogram (ECG) parameters are affected by age [2, 3], and through complex equations, ECG parameters can produce a model of biological age [4, 5]. Similarly, ECG parameters may predict mortality, even in the absence of structural heart diseases, by focusing purely on the person’s age. A number of predictive models for all-cause death using ECG parameters have been reported [6–10] that are based on the concept that abnormal ECG changes represent serious comorbidities that increase the risk of all-cause death. These models have used one or a few parameters, mostly categorical, and were limited to a specific ECG lead [6–10]. Very recently, reported studies have applied machine learning algorithms to large populations and a large numbers of parameters [11].

In this study, we developed predictive models for all-cause death using the 12-lead ECG parameters. Notably, we selected parameters for the models in a stepwise manner from among hundreds of automatically measured ECG parameters. In addition, to prioritize the generalizability of the study focusing on aging, we excluded patients with structural heart diseases to avoid the strong effects these conditions have on mortality.

## Methods

### Study population

The Shinken Database is a single hospital-based database that was established in June 2004 and includes data on all patients newly visiting the Cardiovascular Institute, Tokyo, Japan, excluding foreign travelers and patients with active cancer. Details of this database have been described elsewhere [12].

In the present study, a database of ECG results was used, which has been available since February 2010. From a total of 32,570 patients in the Shinken Database, 19,170 patients registered between February 2010 and March 2018 were extracted. After excluding patients with structural heart diseases (*n* = 4,915); patients aged < 20 years or > 90 years (< 20 or > 90 years; *n* = 168); and patients with index ECG showing indeterminate axis (R axis > 180°) (*n* = 76), pacing beats (*n* = 102), and atrial or ventricular tachyarrhythmia (*n* = 1,763), 12,837 patients were included in the present study. The structural heart diseases were defined as follows: valvular heart disease, moderate or severe stenosis or regurgitation on echocardiography; coronary artery disease; hypertrophic and dilated cardiomyopathy; and symptomatic heart failure [13].

### Patient follow-up

The health status and incidences of cardiovascular events and all-cause death were obtained once per year from the medical records or the postal prognosis documents. In the present study, we included the follow-up data until March 2019 and excluded follow-up data from > 3 years after the initial visit to avoid an imbalance in the follow-up periods as a result of the different registration years (between 2010 and 2018) [13].

### Parameters obtained from ECG

The 12-lead ECG was recorded by a GE ECG machine (GE CardioSoft V6.71 and MAC 5500 HD; GE Healthcare, Chicago, IL), and data were stored using the MUSE data management system [13]. Of the 639 parameters that had been automatically measured, 201 parameters (of which 9 were not lead-specific and 192 [16 × 12 leads] were lead-specific), including the relative coordinate points (i.e., the start point of P-wave), were excluded from the analysis [13]. Accordingly, the remaining 438 parameters (of which 6 were not lead-specific and 432 [36 × 12 leads] were lead-specific) were used in the analysis (Table 1).

**Table 1:** ECG parameters used in this study

Parameters available in MUSE database system: 639 parameters
Parameters used for analysis: 438 parameters
(1) Non-lead-specific parameters: 6 parameters P-R Interval, P axis, QRS Duration, QTc Calculation (QTc Bazett), R axis, T axis (2) Lead-specific parameters: 432 [36 × 12 leads] parameters ST at J Point, P Area, P′ Area, P Area (Full), P Peak Time, P′ Peak Time, P Peak Amplitude, P′ Peak Amplitude, P Duration, P′ Duration, QRS Area, Q Area, Q Peak Amplitude, Q Duration, R Area, R′ Area, R Peak Time, R Duration, R′ Duration, S Area, S′ Area, S Peak Time, S Duration, S′ Duration, T Area, T′ Area, T Area (Full), T Peak Time, T Peak Amplitude, T′ Peak Amplitude, T Duration, T′ Duration, Minimum ST level, Max R Amplitude, Maximum ST level, Max S Amplitude
Parameters excluded: 201 parameters
(1) Non-lead-specific parameters: 9 parameters P Onset, P Offset, QRS Count, QTc Framingham, QTc Fridercia, Q-T Interval, Q Onset, Q Offset, T Offset (2) Lead-specific parameters: 192 [16 × 12 leads] parameters P Onset Amplitude, QRS Balance, QRS Deflection, QRS Intrinsicoid, Q Peak Time, R′ Peak Time, R Peak Amplitude, R′ Peak Amplitude, S′ Peak Time, S Peak Amplitude, S′ Peak Amplitude, T′ Peak Time, T End, ST at End ST, ST at Mid ST, Special T

P′, R′, S′, and T′ indicate the second components of P, R, S, and T wave, respectively, which could be positive or negative polarity

### Evaluation and statistical analysis

Statistical analysis was carried out using SPSS version 26.0 and SPSS Modeler version 18.2 (IBM, Chicago, IL). In all analyses, *P* < 0.05 was taken to indicate statistical significance. Categorical and consecutive data are presented as number (%) and mean ± SD.

**Fig. 1:**
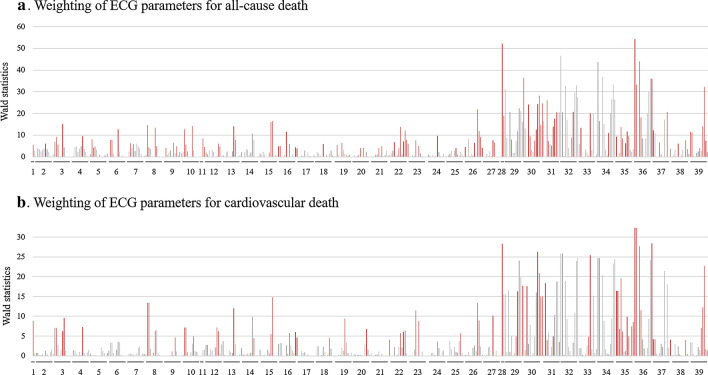
Weighting of predictive capability for all-cause and cardiovascular deaths with 438 ECG parameters. Wald statistics in the univariable logistic regression models for all-cause death (**a**) and cardiovascular death (**b**) with ECG parameters are presented in the order of time-phases. The gray bars indicate the parameters obtained with the MUSE system (438 parameters) and red bars indicate the parameters selected for the final prediction models (109 and 70 parameters for all-cause and cardiovascular death, respectively). The labels under the bars were represented as follows; 1 PR-interval and P axis, 2 P Area, 3 P′ Area, 4 P Area (Full), 5 P Peak Time, 6 P′ Peak Time, 7 P Peak Amplitude, 8 P′ Peak Amplitude, 9 P Duration, 10 P′ Duration, 11 QRS duration and R Axis, 12 QRS Area, 13 Q Area, 14 Q Peak Amplitude, 15 Q Duration, 16 R Area, 17 R′ Area, 18 R Peak Time, 19 Max R Amplitude, 20 R Duration, 21 R′ Duration, 22 S Area, 23 S′ Area, 24 S Peak Time, 25 Max S Amplitude, 26 S Duration, 27 S′ Duration, 28 QTc and T Axis, 29 ST at J Point, 30 Minimum ST level, 31 Maximum ST level, 32 T Area, 33 T′ Area, 34 T Area (Full), 35 T Peak Time, 36 T Peak Amplitude, 37 T′ Peak Amplitude, 38 T Duration, 39 T′ Duration. The order of the lead specific parameter was I, II, III, aVR, aVL, aVF, V1, V2, V3, V4, V5, and V6

We developed a predictive model for all-cause and cardiovascular (CV) death using 438 ECG parameters according to the following steps. *Step 1:* Univariable logistic regression analysis was performed for 438 ECG parameters (Fig. 1a, b; Wald statistics for each parameter are presented). *Step 2:* The Spearman’s coefficient of correlation was evaluated all combinations of the 438 parameters (438 × 437 = 191,406 combinations, excluding the pairing of A vs. A). The parameters combinations with correlation coefficients ≥ 0.9 (defined as “strong correlation”) were identified. Among them, those that demonstrated higher Wald statistics in Step 1 compared with any counterparts were selected for the next step. Parameters were also selected for the next step when they were not included in any pairs with “strong correlation”. *Step 3:* Among the ECG parameters selected in Step 2, parameters with statistical significance in the univariable logistic regression analysis (Wald statistics > 3.841458 [corresponding to *P* < 0.05] in Step 1) were selected for the final model. *Step 4:* Using the ECG parameters selected in Step 3, a prediction model was developed by a support vector machine (SVM) algorithm. For robust evaluation, a tenfold cross-validation method was employed, in which the study patients were divided into 10 similarly-sized groups according to the last digit of their study number (0 to 9), and the model was run 10 times using different combinations of training and testing datasets. For the first run, the testing dataset comprised the group with study numbers ending in 0, and the training dataset comprised the remaining nine groups; for the second run, the testing dataset comprised the group with study numbers ending in 1, and the training dataset comprised the remaining nine groups. The modelling was repeated like so for each of the 10 groups until the testing dataset comprised the group with study numbers ending in 9. The average values of permutation importance [14, 15] for each parameter and the average values of c-statistics were calculated, which evaluated the parameter importance and the model predictive ability, respectively.

For the final results, the following two values were described: (1) Relative importance of ECG parameters. The top 30 values of relative importance (%) were presented, which were calculated using the following equation: [average permutation importance of a parameter] / [average permutation importance of the top 1 parameter] × 100 (%), where the average permutation importance was the average of 10 permutation importance values obtained in the 10 training datasets. The full list of permutation importance values is presented in the Additional file 1: Tables S2 and S3. (2) Predictive capability: the c-statistics represented the predictive capability of the SVM models. C-statistics were separately evaluated for 10 training and 10 testing datasets, and their overall average values with standard deviation were described.

## Results

### Patient characteristics

The characteristics of 12,837 patients are shown in Table 2. The patients in the study included 6,897 males (53.7%), and the mean age of all patients was 55.5 ± 15.0 years.

**Table 2:** Patient characteristics

	Total	Male			Female		
	*n* = 12,837	Total *n* = 6897	Alive *n* = 6863	Deceased *n* = 34	Total *n* = 5940	Alive *n* = 5919	Deceased *n* = 21
Age, years	55.5 ± 15.0	54.2 ± 14.4	54.1 ± 14.4	70.9 ± 12.1	57.0 ± 15.6	56.9 ± 15.6	70.1 ± 14.1
Male, *n* (%)	6897 (53.7)	–	–	–	–	–	–
Body mass index, kg/m^2^	23.4 ± 27.0	24.2 ± 4.5	24.2 ± 4.5	23.3 ± 4.1	22.5 ± 39.3	22.5 ± 39.4	22.5 ± 3.5
Systolic blood pressure, mmHg	125.8 ± 18.5	127.5 ± 16.7	127.5 ± 16.7	125.0 ± 16.9	124.0 ± 20.2	124.0 ± 20.2	131.3 ± 22.0
Diastolic blood pressure, mmHg	75.3 ± 13.8	77.1 ± 11.5	77.1 ± 11.5	71.0 ± 14.0	73.2 ± 15.8	73.2 ± 15.8	74.3 ± 10.3
Heart rate, beats/minute	71.1 ± 12.9	71.2 ± 13.5	71.2 ± 13.4	73.8 ± 17.8	71.0 ± 12.3	71.0 ± 12.3	74.3 ± 17.0
Estimated glomerular filtration rate, mL/min/1.73 m^2^	74.9 ± 17.7	74.3 ± 17.0	74.5 ± 16.9	57.6 ± 23.0	75.5 ± 18.5	75.6 ± 18.4	62.7 ± 29.2
Left ventricular ejection fraction, %	67.8 ± 6.8	66.3 ± 6.6	66.3 ± 6.6	62.8 ± 13.6	69.5 ± 6.5	69.5 ± 6.5	66.1 ± 8.4
Hypertension, *n* (%)	4484 (34.9)	2628 (38.1)	2607 (38.0)	21 (61.8)	1856 (31.2)	1845 (31.2)	11 (52.3)
Dyslipidemia, *n* (%)	2855 (22.2)	1497 (21.7)	1488 (21.7)	9 (26.5)	1358 (22.9)	1353 (22.9)	5 (23.8)
Diabetes, *n* (%)	923 (7.2)	640 (0.9)	631 (9.2)	9 (26.5)	283 (4.8)	278 (4.7)	5 (23.8)
Hyperuricemia, *n* (%)	1362 (10.6)	1160 (16.8)	1149 (16.7)	11 (32.4)	202 (3.4)	199 (3.4)	3 (14.3)
Chronic kidney disease, *n* (%)	1100 (8.6)	617 (8.9)	603 (8.8)	14 (41.2)	483 (8.1)	475 (8.0)	8 (38.1)
Anemia (hemoglobin < 11 g/dL), *n* (%)	186 (1.4)	55 (0.8)	46 (0.7)	9 (26.5)	131 (2.2)	127 (2.1)	4 (19.0)

Consecutive values are presented as mean ± standard deviation

### Incidence of all-cause and CV death

During the mean follow-up period of 320.4 days, all-cause deaths occurred in 55 patients (0.5 per 100 patient-years), among which 23 were cardiovascular deaths (0.2 per 100 patient-years).

### Predictive models for all-cause and CV death

*Step 1:* The Wald statistics of 438 ECG parameters in the univariable logistic regression analysis for all-cause death and CV death are shown in the order of ECG time phases (P, QRS, and ST-T) in Fig. 1a, b, respectively (gray and red bars; the full list is shown in Additional file 1: Table S1). *Step 2:* Spearman’s coefficient of correlation was evaluated for any pairs in the 438 ECG parameters. Among the 438 ECG parameters, 276 parameters did not have combinations with a correlation coefficient ≥ 0.9, thus, they did not have counterparts with a strong correlation. The remaining 162 had combinations with correlation coefficients of ≥ 0.9. Among them, we selected 54 and 52 parameters that had higher Wald statistics compared with any counterparts in Step 1 for all-cause and CV death, respectively. Accordingly, a total of 330 parameters (276 + 54) for all-cause death and 328 parameters (276 + 52) for CV death were selected for the next step. *Step 3:* Among the 330 and 328 parameters selected in Step 3, 109 and 70 parameters with statistical significance in the univariable models for all-cause and CV death, respectively, in Step 1 (Wald statistics > 3.841458) were selected for the final model (Fig. 1a, b, red bars). *Step 4:* Using the respective 109 and 70 parameters, the prediction models for all-cause and CV death were developed by an SVM algorithm. The results are shown below.

## Relative importance of ECG parameters

The permutation importance of the 109 and 70 ECG parameters for all-cause and CV death, respectively, were analyzed in 10 patterns of training datasets by SVM, and their mean values were calculated (Additional file 1: Tables S2 and S3). The top 30 parameters based on the mean permutation importance for all-cause death are listed in Table 3, where T Peak Amplitude in II (100%) demonstrated the highest value, followed by T Peak Amplitude in aVR (60%), T′ Peak Amplitude in aVL (52%), T Peak Amplitude in aVL (50%), and R Peak Time in aVL (50%). The top 30 parameters for CV death are listed in Table 4, where Maximum ST level in I (100%) showed the highest value, followed by S Area in V1 (82%) and Q Duration in V1 (80%).

**Table 3:** The top 30 relative importance (%) for all-cause death

Ranking	Parameter	Relative importance (%)
1	T Peak Amplitude in II	100
2	T Peak Amplitude in aVR	60
3	T′ Peak Amplitude in aVL	52
4	R Peak Time in aVL	50
5	T Peak Amplitude in aVL	50
6	Maximum ST level in V1	48
7	Maximum ST level in V3	48
8	Max R Amplitude in aVL	48
9	QRS Duration	47
10	S Duration in III	47
11	Minimum ST level in aVL	47
12	Max S Amplitude in V1	46
13	S Duration in I	46
14	S Area in aVF	44
15	S Duration in V5	43
16	T′ Area in II	42
17	Maximum ST level in I	42
18	P Area (Full) in V1	40
19	T′ Duration in V5	40
20	S Area in V6	40
21	R Area in V6	39
22	Max R Amplitude in II	39
23	P Peak Time in I	39
24	P′ Peak Time in aVF	38
25	S Area in V5	38
26	T Peak Time in V3	38
27	R Area in V5	38
28	T Peak Time in I	38
29	Minimum ST level in V2	38
30	P Peak Amplitude in aVR	37

**Table 4:** The top 30 relative importance for cardiovascular death

Ranking	Parameters	Relative importance (%)
1	Maximum ST level in I	100
2	S Area in V1	82
3	Q Duration in V1	80
4	Max R Amplitude in V1	79
5	Maximum ST level in II	77
6	R Area in V6	77
7	Q Duration in V2	76
8	T Peak Amplitude in aVL	75
9	R Area in V5	74
10	Maximum ST level in V3	74
11	S Area in V4	73
12	P Area (Full) in V1	73
13	QRS Area in V2	73
14	P′ Duration in V1	72
15	S Duration in V4	72
16	T Duration in V4	72
17	T axis	72
18	QRS Area in V3	72
19	T Peak Time in I	72
20	T Peak Time in V6	71
21	T′ Area in V1	71
22	P Duration in V1	71
23	QTc Calculation (QTc Bazett)	71
24	Max S Amplitude in V4	71
25	S Duration in V3	71
26	T′ Area in V2	71
27	T Peak Time in V3	71
28	T Peak Amplitude in V6	71
29	T Peak Amplitude in II	70
30	T Peak Time in III	70

## Predictive capability

The predictive capability of c-statistics (the mean ± SD of 10 model runs) was 0.881 ± 0.027 in the training dataset and 0.927 ± 0.101 in the testing dataset for all-cause death, and 0.862 ± 0.029 in the training model and 0.897 ± 0.069 in the testing model for CV death. The full list of the 10 combinations of training and testing datasets are provided in Additional file 1: Table S4.

## Discussion

### Major outcomes

The major outcomes of the present study were as follows: (1) We developed a predictive model for all-cause death, in which the mean c-statistics of 10 model runs were 0.881 ± 0.027 for the training dataset and 0.927 ± 0.101 for the testing dataset. (2) The mean c-statistics of 10 model runs for CV death were 0.862 ± 0.029 for the training dataset and 0.897 ± 0.069 for the testing dataset. (3) ECG parameters with high permutation importance for both all-cause and CV death were concentrated in the QRS complex and ST-T segment.

### Comparison with previous studies and clinical implications of our model

Several studies have investigated the feasibility of using visible ECG parameters, including P wave characteristics [8,^ 16^, 17], QRS morphologies [18, 19], ST-T segments, [9, 10] or QT duration, to predict mortality [20]. In a previous study, several ECG parameters, including heart rate > 75 bpm, QRS transition zone > V4, left ventricular hypertrophy, frontal QRS-T angle > 90°, prolonged QTc interval, and prolonged Tpeak-to-Tend interval, were identified as predictors of sudden cardiac death [6]. Our data were partially consistent with this previous study because the ECG parameters that potentially contributed to the prediction of all-cause and CV death were widely distributed among the P, QRS, and ST-T segment. However, when we comprehensively analyzed the ECG parameters contributing to the prediction of all-cause and CV death based on Wald statistics or parameter importance, the most important parameters were concentrated in the QRS complex and ST-T segment for both outcomes. Although the risk models in our study and the previous study [6] were similar to some extent, it is natural that the studies had different findings, as the former predicted all-cause and CV death in patients without structural heart diseases, whereas the latter predicted sudden cardiac death and included patients with structural heart diseases [6].

There have been many studies demonstrating the association between abnormal findings in the QRS complex and ST-T segment and CV mortality. Left ventricular hypertrophy [6] and fragmented QRS [19] have been demonstrated to be risk factors for CV death. The QRS complex is affected by electrophysiological impulse generation and propagation through the ventricles. [21] The pathology underlying QRS abnormality, such as ventricular fibrosis, inflammation, edema, fatty inflammation, ischemic cellular changes, or abnormal myocardial deposition of substances [21], can affect the current or future cardiac function, which may lead to a worse prognosis. The electrocardiographic strain pattern is associated with left ventricular concentric remodeling and scarring. Therefore, it is associated with the future development of various CV events, including heart failure or myocardial infarction, which result in increased mortality, even for patients free from CV diseases at baseline [9].

Our models demonstrated better predictive capability for all-cause death (the mean c-statistics of 10 models was 0.881 ± 0.027 for the training dataset and 0.927 ± 0.101 for the testing dataset) and CV death (0.862 ± 0.029 for the training model and 0.897 ± 0.069 for the testing model) than previous studies that used a few ECG parameters, in which the c-statistics for predicting death were 0.58 (maximal P wave duration) [22], 0.64 (minimal P′ amplitude in lead V1 and V2) [22], 0.61 (QRS area) [23], 0.55 (QRS morphology) [23], 0.51 (QRS duration) [23], 0.727 (QRS-T angle), [24] and 0.759 (model including clinical variables, such as age, sex, hypertension, diabetes, and ECG parameters) [6]. Not surprisingly, the high predictive capabilities of our models are due to the use of a large number of ECG parameters as consecutive values and the application of machine learning, which may sacrifice simplicity but prioritize the predictive capability [11].

### Clinical implications of this study

In this study, we used hundreds of ECG parameters with automatic measurement. Such analysis would have sense when we excluded patients with heart diseases, because the characteristics in ECG with heart diseases are visually apparent. We intended to concentrate on the differences which are visually difficult to be distinguished. We thought small differences in numerical measurement of ECG parameters are affected by age, which should include the atherosclerotic changes in aorta, remodeling in heart, or simply the change of body shapes. For this purpose, we excluded patients with heart diseases, and consequently, the number of the endpoints in the present study (all-cause death or cardiovascular death) became very small.

Further, we should discuss the advantages and disadvantages of using machine learning approach in such prognostic studies. Initially, we intended to develop the clear and practical predictive model or risk score by Cox regression analysis. However, considering the small number of events, the multivariate analysis was clearly oversized in view of the number of events and raised the problem of statistical power. As we could not increase the statistical power (i.e., increase the incidence number of endpoints), we abandoned to use the statistical model like Cox regression analysis. Instead, we employed the machine learning algorithm which can work with a small number of events and a relatively large number of parameters.

Considering the possibility of overfitting by machine learning algorithm, we do not emphasize the differences in the effect of the individual ECG parameters on mortality. However, we believe our data provided a panoramic viewpoint and suggested that the ECG parameters affecting mortality were mostly concentrated in the QRS complex and the ST-T segment.

## Limitations

This study had several limitations. First, all participants in this study were Japanese patients who visited a specialized cardiovascular hospital. Therefore, the results should be interpreted carefully when applied to other populations. Second, we used the parameters measured with a GE ECG machine. The approaches or algorithms to measure the waves may be slightly different between manufacturers of ECG machines, and validation with other ECG machines may be necessary. Third, patient characteristics, such as age, sex, cardiac anatomical information, or concomitant diseases, were not included in our models. Fourth, in the present study, we excluded patients with structural heart diseases. When patients with structural heart diseases are included, the predictive models are more complex and the weight of each ECG parameter for the risk of all-cause death changes. Fifth, we separated the entire cohort into training dataset and testing dataset for developing the models for the purpose of internal validation. However, our model was not validated in an external cohort. Finally, our data did not identify the cutoff values of each parameter nor provide a clear and practical prediction model due to a nature of a machine learning method.

## Conclusion

Parameters acquired from 12-lead resting electrocardiography could be applied to the prediction of all-cause and CV death in patients without structural heart diseases. ECG parameters that greatly contributed to the prediction were concentrated in the QRS complex and ST-T segment.

## Supplementary Information

**Additional file 1: Table S1**. The full list of 438 ECG parameters analyzed by univariate logistic regression analysis. **Table S2**. The permutation importance of the 109 ECG parameters for all-cause death. **Table S3**. The permutation importance of the 109 ECG parameters for cardiovascular death. **Table S4**. The c-statistics of the predictive models for all-cause and cardiovascular death by support vector machine.

## Data Availability

Data cannot be shared publicly because of a lack of such a description in the study protocol and informed consent. Data are available from the Ethics Review Committee at the Cardiovascular Institute for researchers who meet the criteria for access to confidential data (contact via the corresponding author).

## Abbreviations

ECG: Electrocardiogram
NYHA: New York Heart Association
CV: Cardiovascular
SVM: Support vector machine
