# Supplementary material for: Relationship between resting 12-lead electrocardiogram and all-cause death in patients without structural heart disease: Shinken Database analysis
Source: BMC Cardiovasc Disord. 2021 Feb 10;21:83. doi: 10.1186/s12872-021-01864-3 (PMC7874456; doi:10.1186/s12872-021-01864-3)
Supplement: Supplementary file 1 — Additional file 1: Table S1. The full list of 438 ECG parameters analyzed by univariate logistic regression analysis. Table S2. The permutation importance of the 109 ECG parameters for all-cause death. Table S3. The permutation importance of the 109 ECG parameters for cardiovascular death. Table S4. The c-statistics of the predictive models for all-cause and cardiovascular death by support vector machine. [file 12872_2021_1864_MOESM1_ESM.docx]

**Additional file 1: Table S1. The full list of 438 ECG parameters analyzed by univariate logistic regression analysis**

| Parameters | All-cause death | | Cardiovascular death | |
| --- | --- | --- | --- | --- |
|  | Wald statistics | P value | Wald statistics | P value |
| P-R Interval | 12.061 | 0.001 | 8.839 | 0.003 |
| P axis | 1.009 | 0.315 | 0.313 | 0.576 |
| QRS Duration | 2.454 | 0.117 | 1.347 | 0.246 |
| QTc Calculation (QTc Bazett) | 4.433 | 0.035 | 28.375 | <0.001 |
| R axis | 0.057 | 0.812 | 1.501 | 0.221 |
| T axis | 0.616 | 0.433 | 3.506 | 0.061 |
| P Area in I | 0.161 | 0.688 | 0.675 | 0.411 |
| P' Area in I | 1.742 | 0.187 | 7.013 | 0.008 |
| P Area (Full) in I | 0.298 | 0.585 | 1.346 | 0.245 |
| P Peak Time in I | 0.606 | 0.436 | 0.113 | 0.736 |
| P' Peak Time in I | 2.479 | 0.115 | 3.292 | 0.069 |
| P Peak Amplitude in I | 1.048 | 0.306 | 0.480 | 0.488 |
| P' Peak Amplitude in I | 2.146 | 0.143 | 13.442 | <0.001 |
| P Duration in I | 8.293 | 0.004 | 0.002 | 0.964 |
| P' Duration in I | 0.653 | 0.419 | 7.138 | 0.007 |
| QRS Area in I | 0.077 | 0.782 | 2.788 | 0.094 |
| Q Area in I | 0.101 | 0.751 | 0.142 | 0.706 |
| Q Peak Amplitude in I | 0.000 | 0.984 | 0.297 | 0.585 |
| Q Duration in I | 0.584 | 0.445 | 0.117 | 0.732 |
| R Area in I | 0.069 | 0.793 | 3.246 | 0.071 |
| R' Area in I | 0.652 | 0.419 | 0.000 | 0.984 |
| R Peak Time in I | 1.800 | 0.180 | 2.401 | 0.121 |
| Max R Amplitude in I | 0.026 | 0.872 | 0.035 | 0.852 |
| R Duration in I | 0.001 | 0.977 | 0.407 | 0.523 |
| R' Duration in I | 0.495 | 0.482 | 0.000 | 0.993 |
| S Area in I | 1.024 | 0.312 | 0.077 | 0.781 |
| S' Area in I | 0.000 | 0.996 | 0.000 | 0.996 |
| S Peak Time in I | 0.074 | 0.786 | 0.001 | 0.969 |
| Max S Amplitude in I | 1.158 | 0.282 | 0.007 | 0.935 |
| S Duration in I | 1.078 | 0.299 | 0.253 | 0.615 |
| S' Duration in I | 0.000 | 0.999 | 0.000 | 0.998 |
| ST at J Point in I | 9.581 | 0.002 | 15.511 | <0.001 |
| Minimum ST level in I | 11.890 | 0.001 | 17.713 | <0.001 |
| Maximum ST level in I | 7.561 | 0.006 | 14.968 | <0.001 |
| T Area in I | 6.158 | 0.013 | 25.767 | <0.001 |
| T' Area in I | 4.553 | 0.033 | 1.934 | 0.164 |
| T Area (Full) in I | 5.804 | 0.016 | 24.708 | <0.001 |
| T Peak Time in I | 0.614 | 0.433 | 16.34 | <0.001 |
| T Peak Amplitude in I | 5.245 | 0.022 | 32.351 | <0.001 |
| T' Peak Amplitude in I | 4.073 | 0.044 | 4.165 | 0.041 |
| T Duration in I | 1.246 | 0.264 | 0.189 | 0.663 |
| T' Duration in I | 10.301 | 0.001 | 3.44 | 0.063 |
| P Area in II | 0.171 | 0.680 | 0.675 | 0.411 |
| P' Area in II | 1.912 | 0.167 | 7.013 | 0.008 |
| P Area (Full) in II | 0.353 | 0.553 | 1.346 | 0.245 |
| P Peak Time in II | 2.626 | 0.105 | 0.113 | 0.736 |
| P' Peak Time in II | 9.940 | 0.002 | 3.292 | 0.069 |
| P Peak Amplitude in II | 2.194 | 0.139 | 0.480 | 0.488 |
| P' Peak Amplitude in II | 2.404 | 0.121 | 13.442 | <0.001 |
| P Duration in II | 12.283 | <0.001 | 0.002 | 0.964 |
| P' Duration in II | 2.968 | 0.085 | 7.138 | 0.007 |
| QRS Area in II | 0.074 | 0.786 | 2.788 | 0.094 |
| Q Area in II | 0.205 | 0.651 | 0.142 | 0.706 |
| Q Peak Amplitude in II | 1.063 | 0.303 | 0.297 | 0.585 |
| Q Duration in II | 0.018 | 0.892 | 0.117 | 0.732 |
| R Area in II | 0.444 | 0.505 | 3.246 | 0.071 |
| R' Area in II | 0.000 | 0.986 | 0.000 | 0.984 |
| R Peak Time in II | 1.721 | 0.190 | 2.401 | 0.121 |
| Max R Amplitude in II | 3.679 | 0.055 | 0.035 | 0.852 |
| R Duration in II | 2.949 | 0.086 | 0.407 | 0.523 |
| R' Duration in II | 4.216 | 0.040 | 0.000 | 0.993 |
| S Area in II | 0.436 | 0.509 | 0.077 | 0.781 |
| S' Area in II | 0.000 | 0.910 | 0.000 | 0.996 |
| S Peak Time in II | 0.623 | 0.430 | 0.001 | 0.969 |
| Max S Amplitude in II | 3.752 | 0.053 | 0.007 | 0.935 |
| S Duration in II | 1.198 | 0.274 | 0.253 | 0.615 |
| S' Duration in II | 5.992 | 0.014 | 0.000 | 0.998 |
| ST at J Point in II | 0.011 | 0.917 | 15.511 | <0.001 |
| Minimum ST level in II | 0.895 | 0.344 | 17.713 | <0.001 |
| Maximum ST level in II | 2.226 | 0.136 | 14.968 | <0.001 |
| T Area in II | 4.714 | 0.030 | 25.767 | <0.001 |
| T' Area in II | 2.218 | 0.136 | 1.934 | 0.164 |
| T Area (Full) in II | 4.497 | 0.034 | 24.708 | <0.001 |
| T Peak Time in II | 1.140 | 0.286 | 16.34 | <0.001 |
| T Peak Amplitude in II | 3.528 | 0.060 | 32.351 | <0.001 |
| T' Peak Amplitude in II | 3.001 | 0.083 | 4.165 | 0.041 |
| T Duration in II | 0.001 | 0.970 | 0.189 | 0.663 |
| T' Duration in II | 2.384 | 0.123 | 3.44 | 0.063 |
| P Area in III | 0.118 | 0.731 | 0.156 | 0.692 |
| P' Area in III | 0.041 | 0.840 | 2.759 | 0.096 |
| P Area (Full) in III | 0.084 | 0.772 | 0.452 | 0.501 |
| P Peak Time in III | 1.092 | 0.296 | 0.141 | 0.707 |
| P' Peak Time in III | 7.581 | 0.006 | 0.725 | 0.394 |
| P Peak Amplitude in III | 0.000 | 0.995 | 0.498 | 0.480 |
| P' Peak Amplitude in III | 1.006 | 0.316 | 1.865 | 0.172 |
| P Duration in III | 1.169 | 0.280 | 0.061 | 0.804 |
| P' Duration in III | 4.313 | 0.038 | 0.871 | 0.350 |
| QRS Area in III | 0.004 | 0.950 | 0.311 | 0.576 |
| Q Area in III | 0.516 | 0.473 | 0.015 | 0.903 |
| Q Peak Amplitude in III | 0.598 | 0.439 | 0.31 | 0.577 |
| Q Duration in III | 0.020 | 0.887 | 0.346 | 0.556 |
| R Area in III | 0.000 | 0.992 | 0.005 | 0.941 |
| R' Area in III | 1.290 | 0.256 | 1.129 | 0.287 |
| R Peak Time in III | 0.109 | 0.741 | 0.009 | 0.924 |
| Max R Amplitude in III | 0.610 | 0.435 | 0.317 | 0.573 |
| R Duration in III | 0.004 | 0.951 | 0.212 | 0.645 |
| R' Duration in III | 0.596 | 0.440 | 0.218 | 0.640 |
| S Area in III | 1.284 | 0.257 | 2.239 | 0.134 |
| S' Area in III | 0.000 | 0.501 | 0.034 | 0.853 |
| S Peak Time in III | 1.539 | 0.215 | 0.702 | 0.402 |
| Max S Amplitude in III | 0.040 | 0.841 | 3.735 | 0.053 |
| S Duration in III | 0.365 | 0.546 | 0.898 | 0.343 |
| S' Duration in III | 0.162 | 0.687 | 0.338 | 0.561 |
| ST at J Point in III | 4.124 | 0.042 | 0.938 | 0.332 |
| Minimum ST level in III | 0.127 | 0.722 | 1.443 | 0.229 |
| Maximum ST level in III | 1.476 | 0.224 | 0.199 | 0.655 |
| T Area in III | 0.256 | 0.613 | 0.254 | 0.614 |
| T' Area in III | 1.831 | 0.176 | 0.242 | 0.622 |
| T Area (Full) in III | 0.129 | 0.720 | 0.257 | 0.612 |
| T Peak Time in III | 5.844 | 0.016 | 6.746 | 0.009 |
| T Peak Amplitude in III | 0.282 | 0.595 | 0.043 | 0.835 |
| T' Peak Amplitude in III | 1.744 | 0.187 | 0.504 | 0.477 |
| T Duration in III | 3.752 | 0.053 | 3.026 | 0.081 |
| T' Duration in III | 0.903 | 0.342 | 0.409 | 0.522 |
| P Area in aVR | 0.428 | 0.513 | 0.205 | 0.650 |
| P' Area in aVR | 3.264 | 0.071 | 0.028 | 0.867 |
| P Area (Full) in aVR | 0.581 | 0.446 | 0.163 | 0.686 |
| P Peak Time in aVR | 0.653 | 0.419 | 0.011 | 0.918 |
| P' Peak Time in aVR | 14.207 | <0.001 | 0.000 | 0.995 |
| P Peak Amplitude in aVR | 4.166 | 0.041 | 0.218 | 0.640 |
| P' Peak Amplitude in aVR | 3.949 | 0.047 | 0.021 | 0.885 |
| P Duration in aVR | 20.952 | <0.001 | 0.039 | 0.843 |
| P' Duration in aVR | 11.449 | 0.001 | 0.000 | 0.996 |
| QRS Area in aVR | 0.132 | 0.716 | 1.728 | 0.188 |
| Q Area in aVR | 0.413 | 0.520 | 1.343 | 0.246 |
| Q Peak Amplitude in aVR | 0.069 | 0.793 | 0.489 | 0.484 |
| Q Duration in aVR | 2.756 | 0.097 | 0.001 | 0.978 |
| R Area in aVR | 1.541 | 0.214 | 0.007 | 0.931 |
| R' Area in aVR | 0.466 | 0.495 | 0.747 | 0.387 |
| R Peak Time in aVR | 1.937 | 0.164 | 0.269 | 0.604 |
| Max R Amplitude in aVR | 3.757 | 0.053 | 0.132 | 0.716 |
| R Duration in aVR | 2.429 | 0.119 | 0.232 | 0.630 |
| R' Duration in aVR | 2.432 | 0.119 | 0.293 | 0.588 |
| S Area in aVR | 1.556 | 0.212 | 0.001 | 0.976 |
| S' Area in aVR | 0.000 | 0.120 | 1.551 | 0.212 |
| S Peak Time in aVR | 0.142 | 0.706 | 0.787 | 0.374 |
| Max S Amplitude in aVR | 2.615 | 0.106 | 1.679 | 0.195 |
| S Duration in aVR | 1.622 | 0.203 | 0.173 | 0.677 |
| S' Duration in aVR | 2.130 | 0.144 | 2.093 | 0.147 |
| ST at J Point in aVR | 1.646 | 0.200 | 16.585 | <0.001 |
| Minimum ST level in aVR | 6.074 | 0.014 | 17.596 | <0.001 |
| Maximum ST level in aVR | 5.219 | 0.022 | 18.305 | <0.001 |
| T Area in aVR | 7.149 | 0.008 | 18.94 | <0.001 |
| T' Area in aVR | 4.393 | 0.036 | 0.048 | 0.826 |
| T Area (Full) in aVR | 7.081 | 0.008 | 20.392 | <0.001 |
| T Peak Time in aVR | 2.257 | 0.133 | 19.595 | <0.001 |
| T Peak Amplitude in aVR | 5.503 | 0.019 | 27.683 | <0.001 |
| T' Peak Amplitude in aVR | 4.057 | 0.044 | 0.035 | 0.852 |
| T Duration in aVR | 0.024 | 0.877 | 0.357 | 0.549 |
| T' Duration in aVR | 3.559 | 0.059 | 0.000 | 0.993 |
| P Area in aVL | 1.203 | 0.273 | 0.487 | 0.485 |
| P' Area in aVL | 9.246 | 0.002 | 1.451 | 0.228 |
| P Area (Full) in aVL | 0.001 | 0.976 | 0.941 | 0.332 |
| P Peak Time in aVL | 3.026 | 0.082 | 0.015 | 0.902 |
| P' Peak Time in aVL | 6.754 | 0.009 | 1.569 | 0.210 |
| P Peak Amplitude in aVL | 1.134 | 0.287 | 0.184 | 0.668 |
| P' Peak Amplitude in aVL | 4.998 | 0.025 | 0.723 | 0.395 |
| P Duration in aVL | 1.593 | 0.207 | 1.058 | 0.303 |
| P' Duration in aVL | 7.019 | 0.008 | 0.91 | 0.340 |
| QRS Area in aVL | 0.006 | 0.940 | 1.221 | 0.269 |
| Q Area in aVL | 0.247 | 0.619 | 0.927 | 0.335 |
| Q Peak Amplitude in aVL | 0.020 | 0.889 | 0.429 | 0.512 |
| Q Duration in aVL | 0.002 | 0.969 | 1.504 | 0.220 |
| R Area in aVL | 1.079 | 0.299 | 2.703 | 0.100 |
| R' Area in aVL | 2.767 | 0.096 | 0.882 | 0.347 |
| R Peak Time in aVL | 0.144 | 0.705 | 2.269 | 0.131 |
| Max R Amplitude in aVL | 0.819 | 0.365 | 2.043 | 0.152 |
| R Duration in aVL | 1.170 | 0.279 | 0.259 | 0.610 |
| R' Duration in aVL | 4.011 | 0.045 | 1.074 | 0.299 |
| S Area in aVL | 1.700 | 0.192 | 0.32 | 0.571 |
| S' Area in aVL | 0.000 | 0.891 | 11.392 | <0.001 |
| S Peak Time in aVL | 0.377 | 0.539 | 0.243 | 0.622 |
| Max S Amplitude in aVL | 0.549 | 0.459 | 0.019 | 0.890 |
| S Duration in aVL | 1.614 | 0.204 | 0.065 | 0.798 |
| S' Duration in aVL | 0.452 | 0.501 | 2.356 | 0.124 |
| ST at J Point in aVL | 10.644 | 0.001 | 0.956 | 0.328 |
| Minimum ST level in aVL | 10.996 | 0.001 | 3.061 | 0.080 |
| Maximum ST level in aVL | 3.814 | 0.051 | 4.012 | 0.045 |
| T Area in aVL | 1.636 | 0.201 | 9.328 | 0.002 |
| T' Area in aVL | 0.364 | 0.546 | 0.464 | 0.495 |
| T Area (Full) in aVL | 1.015 | 0.314 | 8.78 | 0.003 |
| T Peak Time in aVL | 0.198 | 0.657 | 6.140 | 0.013 |
| T Peak Amplitude in aVL | 2.120 | 0.145 | 11.57 | <0.001 |
| T' Peak Amplitude in aVL | 0.105 | 0.746 | 0.662 | 0.415 |
| T Duration in aVL | 0.429 | 0.512 | 2.34 | 0.126 |
| T' Duration in aVL | 4.571 | 0.033 | 0.171 | 0.679 |
| P Area in aVF | 0.110 | 0.741 | 0.053 | 0.817 |
| P' Area in aVF | 0.784 | 0.376 | 6.233 | 0.012 |
| P Area (Full) in aVF | 0.199 | 0.655 | 0.177 | 0.674 |
| P Peak Time in aVF | 0.983 | 0.321 | 0.054 | 0.816 |
| P' Peak Time in aVF | 14.143 | <0.001 | 3.528 | 0.060 |
| P Peak Amplitude in aVF | 0.382 | 0.536 | 0.621 | 0.430 |
| P' Peak Amplitude in aVF | 0.002 | 0.969 | 6.262 | 0.012 |
| P Duration in aVF | 5.492 | 0.019 | 0.265 | 0.606 |
| P' Duration in aVF | 6.405 | 0.011 | 3.071 | 0.079 |
| QRS Area in aVF | 0.028 | 0.866 | 0.005 | 0.942 |
| Q Area in aVF | 0.541 | 0.462 | 0.726 | 0.394 |
| Q Peak Amplitude in aVF | 0.995 | 0.319 | 0.753 | 0.385 |
| Q Duration in aVF | 0.048 | 0.826 | 0.592 | 0.441 |
| R Area in aVF | 0.178 | 0.673 | 0.388 | 0.533 |
| R' Area in aVF | 0.036 | 0.850 | 0.063 | 0.801 |
| R Peak Time in aVF | 0.895 | 0.344 | 0.903 | 0.342 |
| Max R Amplitude in aVF | 2.800 | 0.094 | 1.24 | 0.265 |
| R Duration in aVF | 0.115 | 0.735 | 0.139 | 0.709 |
| R' Duration in aVF | 1.361 | 0.243 | 0.093 | 0.760 |
| S Area in aVF | 0.083 | 0.774 | 2.253 | 0.133 |
| S' Area in aVF | 0.000 | 0.370 | 0.001 | 0.970 |
| S Peak Time in aVF | 0.051 | 0.822 | 0.180 | 0.671 |
| Max S Amplitude in aVF | 0.690 | 0.406 | 2.185 | 0.139 |
| S Duration in aVF | 0.201 | 0.654 | 1.995 | 0.157 |
| S' Duration in aVF | 1.320 | 0.251 | 0.000 | 0.986 |
| ST at J Point in aVF | 1.152 | 0.283 | 5.003 | 0.025 |
| Minimum ST level in aVF | 0.251 | 0.616 | 7.905 | 0.004 |
| Maximum ST level in aVF | 0.135 | 0.714 | 5.908 | 0.015 |
| T Area in aVF | 1.887 | 0.170 | 2.209 | 0.137 |
| T' Area in aVF | 1.123 | 0.289 | 2.682 | 0.101 |
| T Area (Full) in aVF | 1.817 | 0.178 | 1.640 | 0.200 |
| T Peak Time in aVF | 0.001 | 0.977 | 0.994 | 0.318 |
| T Peak Amplitude in aVF | 1.375 | 0.241 | 4.114 | 0.042 |
| T' Peak Amplitude in aVF | 1.668 | 0.197 | 3.033 | 0.081 |
| T Duration in aVF | 0.192 | 0.661 | 0.295 | 0.587 |
| T' Duration in aVF | 0.744 | 0.388 | 2.157 | 0.141 |
| P Area in V1 | 7.210 | 0.007 | 1.290 | 0.256 |
| P' Area in V1 | 1.846 | 0.174 | 9.529 | 0.002 |
| P Area (Full) in V1 | 1.390 | 0.238 | 7.265 | 0.007 |
| P Peak Time in V1 | 7.193 | 0.007 | 2.216 | 0.136 |
| P' Peak Time in V1 | 4.183 | 0.041 | 3.414 | 0.064 |
| P Peak Amplitude in V1 | 8.279 | 0.004 | 2.03 | 0.154 |
| P' Peak Amplitude in V1 | 0.216 | 0.642 | 6.468 | 0.010 |
| P Duration in V1 | 1.794 | 0.180 | 4.626 | 0.031 |
| P' Duration in V1 | 2.551 | 0.110 | 4.914 | 0.026 |
| QRS Area in V1 | 0.643 | 0.423 | 2.133 | 0.144 |
| Q Area in V1 | 0.026 | 0.872 | 11.959 | <0.001 |
| Q Peak Amplitude in V1 | 0.027 | 0.871 | 9.807 | 0.001 |
| Q Duration in V1 | 0.057 | 0.812 | 5.468 | 0.019 |
| R Area in V1 | 1.982 | 0.159 | 5.701 | 0.016 |
| R' Area in V1 | 0.551 | 0.458 | 0.387 | 0.533 |
| R Peak Time in V1 | 0.473 | 0.491 | 0.071 | 0.789 |
| Max R Amplitude in V1 | 1.005 | 0.316 | 9.415 | 0.002 |
| R Duration in V1 | 0.147 | 0.702 | 0.067 | 0.795 |
| R' Duration in V1 | 1.623 | 0.203 | 0.482 | 0.487 |
| S Area in V1 | 1.689 | 0.194 | 5.738 | 0.016 |
| S' Area in V1 | 0.000 | 0.295 | 8.737 | 0.003 |
| S Peak Time in V1 | 2.260 | 0.133 | 3.605 | 0.057 |
| Max S Amplitude in V1 | 3.954 | 0.047 | 0.95 | 0.329 |
| S Duration in V1 | 0.008 | 0.929 | 2.559 | 0.109 |
| S' Duration in V1 | 5.869 | 0.015 | 10.139 | 0.001 |
| ST at J Point in V1 | 0.440 | 0.507 | 0.041 | 0.838 |
| Minimum ST level in V1 | 0.662 | 0.416 | 0.026 | 0.872 |
| Maximum ST level in V1 | 0.452 | 0.501 | 0.180 | 0.671 |
| T Area in V1 | 0.266 | 0.606 | 1.187 | 0.275 |
| T' Area in V1 | 3.059 | 0.080 | 4.824 | 0.028 |
| T Area (Full) in V1 | 0.053 | 0.817 | 0.526 | 0.468 |
| T Peak Time in V1 | 0.032 | 0.858 | 1.138 | 0.286 |
| T Peak Amplitude in V1 | 0.348 | 0.555 | 1.802 | 0.179 |
| T' Peak Amplitude in V1 | 1.894 | 0.169 | 2.231 | 0.135 |
| T Duration in V1 | 0.307 | 0.579 | 0.113 | 0.736 |
| T' Duration in V1 | 0.966 | 0.326 | 1.487 | 0.222 |
| P Area in V2 | 7.597 | 0.006 | 0.349 | 0.554 |
| P' Area in V2 | 0.000 | 0.999 | 1.308 | 0.252 |
| P Area (Full) in V2 | 5.579 | 0.018 | 0.611 | 0.434 |
| P Peak Time in V2 | 1.914 | 0.167 | 1.273 | 0.259 |
| P' Peak Time in V2 | 1.435 | 0.231 | 0.281 | 0.595 |
| P Peak Amplitude in V2 | 11.379 | 0.001 | 2.381 | 0.122 |
| P' Peak Amplitude in V2 | 0.559 | 0.455 | 0.559 | 0.454 |
| P Duration in V2 | 0.284 | 0.594 | 1.084 | 0.297 |
| P' Duration in V2 | 1.210 | 0.271 | 1.178 | 0.277 |
| QRS Area in V2 | 0.009 | 0.923 | 7.100 | 0.007 |
| Q Area in V2 | 0.184 | 0.668 | 2.961 | 0.085 |
| Q Peak Amplitude in V2 | 0.096 | 0.756 | 4.436 | 0.035 |
| Q Duration in V2 | 0.177 | 0.674 | 14.825 | <0.001 |
| R Area in V2 | 0.027 | 0.870 | 2.563 | 0.109 |
| R' Area in V2 | 0.003 | 0.957 | 0.005 | 0.943 |
| R Peak Time in V2 | 1.835 | 0.176 | 0.387 | 0.533 |
| Max R Amplitude in V2 | 0.725 | 0.394 | 3.381 | 0.065 |
| R Duration in V2 | 0.918 | 0.338 | 3.066 | 0.079 |
| R' Duration in V2 | 0.259 | 0.611 | 0.000 | 0.982 |
| S Area in V2 | 0.001 | 0.982 | 2.131 | 0.144 |
| S' Area in V2 | 0.000 | 0.912 | 0.001 | 0.974 |
| S Peak Time in V2 | 1.343 | 0.246 | 1.923 | 0.165 |
| Max S Amplitude in V2 | 0.516 | 0.472 | 0.787 | 0.375 |
| S Duration in V2 | 1.097 | 0.295 | 1.197 | 0.274 |
| S' Duration in V2 | 0.010 | 0.921 | 0.000 | 0.992 |
| ST at J Point in V2 | 2.629 | 0.105 | 0.711 | 0.399 |
| Minimum ST level in V2 | 2.485 | 0.115 | 0.645 | 0.421 |
| Maximum ST level in V2 | 2.207 | 0.137 | 2.335 | 0.126 |
| T Area in V2 | 0.493 | 0.483 | 0.030 | 0.861 |
| T' Area in V2 | 0.685 | 0.408 | 25.451 | <0.001 |
| T Area (Full) in V2 | 0.615 | 0.433 | 0.303 | 0.581 |
| T Peak Time in V2 | 2.193 | 0.139 | 9.851 | 0.001 |
| T Peak Amplitude in V2 | 0.603 | 0.437 | 0.025 | 0.875 |
| T' Peak Amplitude in V2 | 0.234 | 0.628 | 21.398 | <0.001 |
| T Duration in V2 | 0.344 | 0.557 | 0.076 | 0.783 |
| T' Duration in V2 | 0.179 | 0.672 | 7.045 | 0.007 |
| P Area in V3 | 6.654 | 0.010 | 0.003 | 0.956 |
| P' Area in V3 | 0.838 | 0.360 | 0.05 | 0.823 |
| P Area (Full) in V3 | 6.876 | 0.009 | 0.001 | 0.981 |
| P Peak Time in V3 | 2.373 | 0.123 | 0.706 | 0.400 |
| P' Peak Time in V3 | 4.649 | 0.031 | 0.018 | 0.891 |
| P Peak Amplitude in V3 | 5.112 | 0.024 | 0.041 | 0.839 |
| P' Peak Amplitude in V3 | 2.173 | 0.140 | 0.120 | 0.728 |
| P Duration in V3 | 7.292 | 0.007 | 0.031 | 0.859 |
| P' Duration in V3 | 6.936 | 0.008 | 0.956 | 0.328 |
| QRS Area in V3 | 0.182 | 0.670 | 6.175 | 0.012 |
| Q Area in V3 | 0.099 | 0.754 | 0.022 | 0.882 |
| Q Peak Amplitude in V3 | 0.127 | 0.721 | 0.003 | 0.956 |
| Q Duration in V3 | 0.239 | 0.625 | 0.119 | 0.729 |
| R Area in V3 | 0.018 | 0.893 | 1.261 | 0.261 |
| R' Area in V3 | 0.406 | 0.524 | 0.009 | 0.923 |
| R Peak Time in V3 | 0.533 | 0.465 | 4.452 | 0.034 |
| Max R Amplitude in V3 | 0.004 | 0.947 | 0.678 | 0.410 |
| R Duration in V3 | 0.318 | 0.573 | 6.790 | 0.009 |
| R' Duration in V3 | 0.747 | 0.388 | 0.079 | 0.778 |
| S Area in V3 | 0.467 | 0.494 | 6.012 | 0.014 |
| S' Area in V3 | 0.000 | 0.433 | 1.038 | 0.308 |
| S Peak Time in V3 | 3.862 | 0.049 | 0.015 | 0.903 |
| Max S Amplitude in V3 | 0.260 | 0.610 | 3.792 | 0.051 |
| S Duration in V3 | 0.652 | 0.419 | 13.327 | <0.001 |
| S' Duration in V3 | 2.668 | 0.102 | 1.233 | 0.266 |
| ST at J Point in V3 | 3.626 | 0.057 | 4.890 | 0.027 |
| Minimum ST level in V3 | 3.503 | 0.061 | 4.771 | 0.028 |
| Maximum ST level in V3 | 3.444 | 0.063 | 4.934 | 0.026 |
| T Area in V3 | 1.295 | 0.255 | 3.32 | 0.068 |
| T' Area in V3 | 0.388 | 0.533 | 1.630 | 0.201 |
| T Area (Full) in V3 | 1.154 | 0.283 | 3.077 | 0.079 |
| T Peak Time in V3 | 1.705 | 0.192 | 4.911 | 0.026 |
| T Peak Amplitude in V3 | 1.168 | 0.280 | 1.965 | 0.160 |
| T' Peak Amplitude in V3 | 0.470 | 0.493 | 0.091 | 0.763 |
| T Duration in V3 | 0.366 | 0.545 | 0.007 | 0.932 |
| T' Duration in V3 | 1.138 | 0.286 | 12.29 | <0.001 |
| P Area in V4 | 6.954 | 0.008 | 0.862 | 0.353 |
| P' Area in V4 | 0.028 | 0.866 | 0.006 | 0.936 |
| P Area (Full) in V4 | 7.267 | 0.007 | 0.826 | 0.363 |
| P Peak Time in V4 | 1.575 | 0.209 | 0.001 | 0.974 |
| P' Peak Time in V4 | 5.739 | 0.017 | 0.000 | 0.993 |
| P Peak Amplitude in V4 | 1.234 | 0.267 | 0.519 | 0.471 |
| P' Peak Amplitude in V4 | 0.009 | 0.925 | 0.045 | 0.832 |
| P Duration in V4 | 9.950 | 0.002 | 0.025 | 0.873 |
| P' Duration in V4 | 9.585 | 0.002 | 0.000 | 0.993 |
| QRS Area in V4 | 0.041 | 0.839 | 0.586 | 0.443 |
| Q Area in V4 | 0.024 | 0.876 | 0.003 | 0.959 |
| Q Peak Amplitude in V4 | 0.042 | 0.837 | 0.172 | 0.677 |
| Q Duration in V4 | 0.155 | 0.694 | 0.045 | 0.832 |
| R Area in V4 | 0.375 | 0.540 | 0.725 | 0.394 |
| R' Area in V4 | 0.065 | 0.798 | 0.002 | 0.964 |
| R Peak Time in V4 | 3.537 | 0.060 | 1.820 | 0.177 |
| Max R Amplitude in V4 | 0.840 | 0.359 | 0.075 | 0.784 |
| R Duration in V4 | 0.015 | 0.901 | 1.656 | 0.198 |
| R' Duration in V4 | 1.256 | 0.262 | 0.000 | 0.989 |
| S Area in V4 | 0.129 | 0.720 | 6.398 | 0.011 |
| S' Area in V4 | 0.000 | 0.086 | 0.001 | 0.981 |
| S Peak Time in V4 | 4.588 | 0.032 | 1.196 | 0.274 |
| Max S Amplitude in V4 | 0.070 | 0.791 | 5.647 | 0.017 |
| S Duration in V4 | 0.831 | 0.362 | 8.958 | 0.002 |
| S' Duration in V4 | 6.603 | 0.010 | 0.000 | 0.994 |
| ST at J Point in V4 | 7.241 | 0.007 | 16.302 | <0.001 |
| Minimum ST level in V4 | 7.298 | 0.007 | 16.026 | <0.001 |
| Maximum ST level in V4 | 5.183 | 0.023 | 10.303 | 0.001 |
| T Area in V4 | 2.895 | 0.089 | 10.908 | <0.001 |
| T' Area in V4 | 0.108 | 0.743 | 15.197 | <0.001 |
| T Area (Full) in V4 | 2.912 | 0.088 | 9.385 | 0.002 |
| T Peak Time in V4 | 1.238 | 0.266 | 0.148 | 0.700 |
| T Peak Amplitude in V4 | 2.549 | 0.110 | 9.289 | 0.002 |
| T' Peak Amplitude in V4 | 0.197 | 0.657 | 18.066 | <0.001 |
| T Duration in V4 | 0.675 | 0.411 | 4.011 | 0.045 |
| T' Duration in V4 | 7.775 | 0.005 | 22.718 | <0.001 |
| P Area in V5 | 3.925 | 0.048 | 1.615 | 0.203 |
| P' Area in V5 | 0.924 | 0.336 | 0.001 | 0.977 |
| P Area (Full) in V5 | 4.469 | 0.035 | 1.536 | 0.215 |
| P Peak Time in V5 | 0.038 | 0.846 | 0.741 | 0.389 |
| P' Peak Time in V5 | 17.299 | <0.001 | 0.000 | 0.995 |
| P Peak Amplitude in V5 | 0.206 | 0.650 | 0.418 | 0.517 |
| P' Peak Amplitude in V5 | 0.133 | 0.716 | 0.002 | 0.967 |
| P Duration in V5 | 12.824 | <0.001 | 0.001 | 0.973 |
| P' Duration in V5 | 13.066 | <0.001 | 0.000 | 0.996 |
| QRS Area in V5 | 0.330 | 0.566 | 2.895 | 0.088 |
| Q Area in V5 | 0.533 | 0.465 | 0.387 | 0.533 |
| Q Peak Amplitude in V5 | 0.685 | 0.408 | 0.051 | 0.821 |
| Q Duration in V5 | 0.273 | 0.601 | 0.006 | 0.936 |
| R Area in V5 | 0.493 | 0.483 | 5.994 | 0.014 |
| R' Area in V5 | 1.689 | 0.194 | 0.003 | 0.959 |
| R Peak Time in V5 | 0.211 | 0.646 | 0.090 | 0.764 |
| Max R Amplitude in V5 | 0.878 | 0.349 | 0.826 | 0.363 |
| R Duration in V5 | 0.001 | 0.971 | 0.205 | 0.650 |
| R' Duration in V5 | 2.828 | 0.093 | 0.000 | 0.991 |
| S Area in V5 | 0.250 | 0.617 | 0.652 | 0.419 |
| S' Area in V5 | 0.000 | 0.895 | 0.000 | 0.987 |
| S Peak Time in V5 | 0.078 | 0.780 | 1.338 | 0.247 |
| Max S Amplitude in V5 | 0.014 | 0.907 | 0.617 | 0.432 |
| S Duration in V5 | 0.671 | 0.413 | 2.261 | 0.132 |
| S' Duration in V5 | 0.677 | 0.411 | 0.000 | 0.996 |
| ST at J Point in V5 | 7.883 | 0.005 | 24.077 | <0.001 |
| Minimum ST level in V5 | 8.344 | 0.004 | 26.26 | <0.001 |
| Maximum ST level in V5 | 6.590 | 0.010 | 18.672 | <0.001 |
| T Area in V5 | 3.838 | 0.050 | 23.93 | <0.001 |
| T' Area in V5 | 0.020 | 0.887 | 0.727 | 0.393 |
| T Area (Full) in V5 | 4.095 | 0.043 | 23.319 | <0.001 |
| T Peak Time in V5 | 1.947 | 0.163 | 7.401 | 0.006 |
| T Peak Amplitude in V5 | 3.265 | 0.071 | 24.144 | <0.001 |
| T' Peak Amplitude in V5 | 0.003 | 0.955 | 2.018 | 0.155 |
| T Duration in V5 | 0.579 | 0.447 | 0.461 | 0.497 |
| T' Duration in V5 | 6.858 | 0.009 | 1.695 | 0.192 |
| P Area in V6 | 3.348 | 0.067 | 0.401 | 0.526 |
| P' Area in V6 | 7.090 | 0.008 | 0.008 | 0.929 |
| P Area (Full) in V6 | 4.506 | 0.034 | 0.483 | 0.486 |
| P Peak Time in V6 | 1.503 | 0.220 | 1.263 | 0.261 |
| P' Peak Time in V6 | 3.523 | 0.061 | 0.000 | 0.996 |
| P Peak Amplitude in V6 | 0.659 | 0.417 | 0.306 | 0.58 |
| P' Peak Amplitude in V6 | 7.866 | 0.005 | 0.003 | 0.958 |
| P Duration in V6 | 9.901 | 0.002 | 0.352 | 0.553 |
| P' Duration in V6 | 3.395 | 0.065 | 0.000 | 0.997 |
| QRS Area in V6 | 0.003 | 0.957 | 3.674 | 0.055 |
| Q Area in V6 | 0.375 | 0.540 | 1.915 | 0.166 |
| Q Peak Amplitude in V6 | 1.151 | 0.283 | 1.549 | 0.213 |
| Q Duration in V6 | 0.558 | 0.455 | 3.019 | 0.082 |
| R Area in V6 | 0.030 | 0.862 | 4.611 | 0.031 |
| R' Area in V6 | 0.475 | 0.490 | 0.036 | 0.848 |
| R Peak Time in V6 | 2.200 | 0.138 | 0.261 | 0.609 |
| Max R Amplitude in V6 | 0.067 | 0.796 | 0.041 | 0.84 |
| R Duration in V6 | 0.049 | 0.825 | 0.574 | 0.448 |
| R' Duration in V6 | 0.193 | 0.660 | 4.046 | 0.044 |
| S Area in V6 | 0.157 | 0.692 | 0.026 | 0.872 |
| S' Area in V6 | 0.000 | 0.995 | 0.000 | 0.988 |
| S Peak Time in V6 | 0.802 | 0.371 | 0.069 | 0.792 |
| Max S Amplitude in V6 | 1.324 | 0.250 | 0.034 | 0.852 |
| S Duration in V6 | 0.565 | 0.452 | 0.22 | 0.639 |
| S' Duration in V6 | 0.000 | 0.998 | 0.000 | 0.997 |
| ST at J Point in V6 | 3.658 | 0.056 | 19.866 | <0.001 |
| Minimum ST level in V6 | 4.606 | 0.032 | 20.842 | <0.001 |
| Maximum ST level in V6 | 4.445 | 0.035 | 18.824 | <0.001 |
| T Area in V6 | 3.537 | 0.060 | 24.731 | <0.001 |
| T' Area in V6 | 0.963 | 0.327 | 1.728 | 0.188 |
| T Area (Full) in V6 | 3.672 | 0.055 | 24.447 | <0.001 |
| T Peak Time in V6 | 0.145 | 0.703 | 8.552 | 0.003 |
| T Peak Amplitude in V6 | 3.174 | 0.075 | 28.454 | <0.001 |
| T' Peak Amplitude in V6 | 2.318 | 0.128 | 4.051 | 0.044 |
| T Duration in V6 | 0.766 | 0.381 | 0.014 | 0.906 |
| T' Duration in V6 | 2.204 | 0.138 | 1.351 | 0.245 |

**Additional file 1: Table S2. The permutation importance of the 109 ECG parameters for all-cause death**

| Parameters | Model 1 | Model 2 | Model 3 | Model 4 | Model 5 | Model 6 | Model 7 | Model 8 | Model 9 | Model 10 | Mean | SD |
| --- | --- | --- | --- | --- | --- | --- | --- | --- | --- | --- | --- | --- |
| P-R Interval | 0.0095 | 0.0093 | 0.0091 | 0.0093 | 0.0005 | 0.0093 | 0.0093 | 0.0092 | 0.0093 | 0.0083 | 0.0083 | 0.0028 |
| P Area in V1 | 0.0092 | 0.0095 | 0.0095 | 0.0097 | 0.0005 | 0.0088 | 0.0094 | 0.0081 | 0.0084 | 0.0083 | 0.0081 | 0.0027 |
| P' Area in II | 0.0094 | 0.0093 | 0.0091 | 0.0095 | 0.0010 | 0.0094 | 0.0093 | 0.0092 | 0.0094 | 0.0084 | 0.0084 | 0.0026 |
| P' Area in III | 0.0095 | 0.0093 | 0.0089 | 0.0095 | 0.0013 | 0.0093 | 0.0092 | 0.0092 | 0.0094 | 0.0086 | 0.0084 | 0.0025 |
| P' Area in aVF | 0.0095 | 0.0094 | 0.0089 | 0.0098 | 0.0014 | 0.0094 | 0.0093 | 0.0092 | 0.0094 | 0.0084 | 0.0085 | 0.0025 |
| P Area (Full) in V1 | 0.0066 | 0.0089 | 0.0089 | 0.0047 | 0.0134 | 0.0090 | 0.0084 | 0.0074 | 0.0090 | 0.0214 | 0.0098 | 0.0046 |
| P Peak Time in I | 0.0098 | 0.0091 | 0.0186 | 0.0091 | 0.0021 | 0.0094 | 0.0092 | 0.0090 | 0.0093 | 0.0099 | 0.0096 | 0.0039 |
| P Peak Time in II | 0.0094 | 0.0093 | 0.0090 | 0.0103 | 0.0008 | 0.0095 | 0.0094 | 0.0103 | 0.0094 | 0.0094 | 0.0087 | 0.0028 |
| P Peak Time in III | 0.0096 | 0.0095 | 0.0091 | 0.0099 | 0.0028 | 0.0095 | 0.0092 | 0.0090 | 0.0094 | 0.0088 | 0.0087 | 0.0021 |
| P' Peak Time in I | 0.0094 | 0.0093 | 0.0093 | 0.0095 | 0.0010 | 0.0094 | 0.0092 | 0.0092 | 0.0094 | 0.0084 | 0.0084 | 0.0026 |
| P' Peak Time in II | 0.0094 | 0.0093 | 0.0091 | 0.0096 | 0.0012 | 0.0094 | 0.0093 | 0.0092 | 0.0093 | 0.0084 | 0.0084 | 0.0026 |
| P' Peak Time in aVF | 0.0094 | 0.0083 | 0.0111 | 0.0113 | 0.0078 | 0.0094 | 0.0093 | 0.0092 | 0.0094 | 0.0087 | 0.0094 | 0.0011 |
| P Peak Amplitude in II | 0.0094 | 0.0094 | 0.0092 | 0.0081 | 0.0098 | 0.0094 | 0.0093 | 0.0088 | 0.0093 | 0.0085 | 0.0091 | 0.0005 |
| P Peak Amplitude in aVR | 0.0097 | 0.0095 | 0.0091 | 0.0094 | 0.0078 | 0.0093 | 0.0093 | 0.0096 | 0.0094 | 0.0084 | 0.0092 | 0.0006 |
| P' Peak Amplitude in aVF | 0.0095 | 0.0094 | 0.0089 | 0.0065 | 0.0005 | 0.0094 | 0.0092 | 0.0092 | 0.0094 | 0.0083 | 0.0080 | 0.0028 |
| P' Peak Amplitude in I | 0.0094 | 0.0094 | 0.0091 | 0.0095 | 0.0010 | 0.0094 | 0.0092 | 0.0090 | 0.0094 | 0.0084 | 0.0084 | 0.0026 |
| P' Peak Amplitude in II | 0.0094 | 0.0093 | 0.0092 | 0.0095 | 0.0010 | 0.0094 | 0.0093 | 0.0092 | 0.0094 | 0.0084 | 0.0084 | 0.0026 |
| P' Peak Amplitude in V1 | 0.0077 | 0.0093 | 0.0097 | 0.0101 | 0.0005 | 0.0092 | 0.0088 | 0.0097 | 0.0079 | 0.0068 | 0.0080 | 0.0028 |
| P Duration in I | 0.0086 | 0.0096 | 0.0089 | 0.0080 | 0.0005 | 0.0094 | 0.0093 | 0.0092 | 0.0093 | 0.0097 | 0.0083 | 0.0028 |
| P Duration in aVF | 0.0097 | 0.0095 | 0.0090 | 0.0074 | 0.0005 | 0.0095 | 0.0094 | 0.0093 | 0.0094 | 0.0087 | 0.0082 | 0.0028 |
| P Duration in V2 | 0.0095 | 0.0094 | 0.0089 | 0.0092 | 0.0005 | 0.0094 | 0.0095 | 0.0098 | 0.0093 | 0.0085 | 0.0084 | 0.0028 |
| P' Duration in I | 0.0094 | 0.0093 | 0.0092 | 0.0095 | 0.0010 | 0.0094 | 0.0092 | 0.0092 | 0.0094 | 0.0084 | 0.0084 | 0.0026 |
| P' Duration in II | 0.0094 | 0.0093 | 0.0095 | 0.0096 | 0.0011 | 0.0094 | 0.0093 | 0.0092 | 0.0094 | 0.0085 | 0.0085 | 0.0026 |
| P' Duration in aVF | 0.0095 | 0.0093 | 0.0099 | 0.0043 | 0.0005 | 0.0094 | 0.0093 | 0.0092 | 0.0094 | 0.0084 | 0.0079 | 0.0031 |
| QRS Duration | 0.0092 | 0.0092 | 0.0092 | 0.0093 | 0.0332 | 0.0093 | 0.0092 | 0.0092 | 0.0095 | 0.0086 | 0.0116 | 0.0076 |
| R axis | 0.0095 | 0.0094 | 0.0092 | 0.0094 | 0.0005 | 0.0095 | 0.0095 | 0.0092 | 0.0095 | 0.0083 | 0.0084 | 0.0028 |
| QRS Area in V3 | 0.0093 | 0.0094 | 0.0092 | 0.0104 | 0.0005 | 0.0093 | 0.0089 | 0.0093 | 0.0094 | 0.0089 | 0.0085 | 0.0028 |
| QRS Area in V4 | 0.0082 | 0.0086 | 0.0096 | 0.0098 | 0.0122 | 0.0094 | 0.0088 | 0.0067 | 0.0089 | 0.0088 | 0.0091 | 0.0014 |
| Q Area in V1 | 0.0095 | 0.0090 | 0.0089 | 0.0098 | 0.0016 | 0.0094 | 0.0093 | 0.0092 | 0.0094 | 0.0085 | 0.0085 | 0.0024 |
| Q Area in V2 | 0.0094 | 0.0083 | 0.0089 | 0.0095 | 0.0005 | 0.0091 | 0.0092 | 0.0092 | 0.0094 | 0.0084 | 0.0082 | 0.0027 |
| Q Duration in V1 | 0.0096 | 0.0093 | 0.0090 | 0.0118 | 0.0005 | 0.0094 | 0.0093 | 0.0092 | 0.0094 | 0.0084 | 0.0086 | 0.0030 |
| Q Duration in V2 | 0.0098 | 0.0094 | 0.0089 | 0.0095 | 0.0084 | 0.0094 | 0.0092 | 0.0091 | 0.0092 | 0.0072 | 0.0090 | 0.0007 |
| Q Duration in V6 | 0.0094 | 0.0094 | 0.0089 | 0.0092 | 0.0019 | 0.0093 | 0.0094 | 0.0090 | 0.0093 | 0.0084 | 0.0084 | 0.0023 |
| R Area in I | 0.0097 | 0.0093 | 0.0091 | 0.0094 | 0.0006 | 0.0094 | 0.0095 | 0.0101 | 0.0094 | 0.0085 | 0.0085 | 0.0028 |
| R Area in aVL | 0.0094 | 0.0087 | 0.0091 | 0.0092 | 0.0006 | 0.0093 | 0.0095 | 0.0100 | 0.0093 | 0.0086 | 0.0084 | 0.0028 |
| R Area in V1 | 0.0094 | 0.0093 | 0.0089 | 0.0098 | 0.0037 | 0.0094 | 0.0093 | 0.0092 | 0.0092 | 0.0084 | 0.0087 | 0.0018 |
| R Area in V5 | 0.0079 | 0.0069 | 0.0095 | 0.0088 | 0.0115 | 0.0093 | 0.0095 | 0.0149 | 0.0085 | 0.0060 | 0.0093 | 0.0025 |
| R Area in V6 | 0.0090 | 0.0088 | 0.0090 | 0.0072 | 0.0179 | 0.0094 | 0.0092 | 0.0070 | 0.0094 | 0.0092 | 0.0096 | 0.0030 |
| R Peak Time in aVL | 0.0095 | 0.0093 | 0.0092 | 0.0098 | 0.0382 | 0.0093 | 0.0092 | 0.0099 | 0.0094 | 0.0082 | 0.0122 | 0.0091 |
| Max R Amplitude in II | 0.0095 | 0.0093 | 0.0096 | 0.0088 | 0.0141 | 0.0089 | 0.0091 | 0.0096 | 0.0091 | 0.0076 | 0.0096 | 0.0017 |
| Max R Amplitude in aVL | 0.0093 | 0.0092 | 0.0090 | 0.0093 | 0.0350 | 0.0094 | 0.0092 | 0.0102 | 0.0094 | 0.0071 | 0.0117 | 0.0082 |
| R Duration in aVL | 0.0092 | 0.0089 | 0.0090 | 0.0095 | 0.0005 | 0.0092 | 0.0093 | 0.0100 | 0.0090 | 0.0080 | 0.0083 | 0.0028 |
| R Duration in V1 | 0.0088 | 0.0089 | 0.0084 | 0.0064 | 0.0072 | 0.0093 | 0.0091 | 0.0091 | 0.0091 | 0.0102 | 0.0087 | 0.0011 |
| R' Duration in aVL | 0.0094 | 0.0095 | 0.0089 | 0.0120 | 0.0029 | 0.0094 | 0.0092 | 0.0091 | 0.0094 | 0.0084 | 0.0088 | 0.0023 |
| R' Duration in V1 | 0.0092 | 0.0092 | 0.0090 | 0.0092 | 0.0045 | 0.0095 | 0.0092 | 0.0090 | 0.0093 | 0.0084 | 0.0087 | 0.0015 |
| S Area in III | 0.0092 | 0.0094 | 0.0091 | 0.0094 | 0.0005 | 0.0094 | 0.0095 | 0.0099 | 0.0094 | 0.0085 | 0.0084 | 0.0028 |
| S Area in aVF | 0.0094 | 0.0094 | 0.0091 | 0.0083 | 0.0263 | 0.0094 | 0.0090 | 0.0085 | 0.0092 | 0.0085 | 0.0107 | 0.0055 |
| S Area in V1 | 0.0095 | 0.0093 | 0.0091 | 0.0110 | 0.0005 | 0.0094 | 0.0092 | 0.0085 | 0.0094 | 0.0084 | 0.0084 | 0.0029 |
| S Area in V3 | 0.0093 | 0.0093 | 0.0092 | 0.0092 | 0.0016 | 0.0092 | 0.0093 | 0.0090 | 0.0094 | 0.0087 | 0.0084 | 0.0024 |
| S Area in V4 | 0.0094 | 0.0094 | 0.0101 | 0.0104 | 0.0051 | 0.0087 | 0.0093 | 0.0062 | 0.0086 | 0.0087 | 0.0086 | 0.0017 |
| S Area in V5 | 0.0076 | 0.0085 | 0.0071 | 0.0063 | 0.0301 | 0.0042 | 0.0065 | 0.0082 | 0.0081 | 0.0070 | 0.0094 | 0.0074 |
| S Area in V6 | 0.0091 | 0.0094 | 0.0081 | 0.0106 | 0.0160 | 0.0091 | 0.0092 | 0.0090 | 0.0087 | 0.0075 | 0.0097 | 0.0024 |
| S' Area in aVL | 0.0094 | 0.0093 | 0.0089 | 0.0096 | 0.0010 | 0.0094 | 0.0092 | 0.0092 | 0.0094 | 0.0084 | 0.0084 | 0.0026 |
| S' Area in V1 | 0.0095 | 0.0087 | 0.0091 | 0.0096 | 0.0010 | 0.0094 | 0.0092 | 0.0089 | 0.0094 | 0.0085 | 0.0083 | 0.0026 |
| S Peak Time in V1 | 0.0095 | 0.0092 | 0.0091 | 0.0103 | 0.0005 | 0.0093 | 0.0092 | 0.0086 | 0.0092 | 0.0080 | 0.0083 | 0.0028 |
| Max S Amplitude in V1 | 0.0097 | 0.0090 | 0.0093 | 0.0101 | 0.0265 | 0.0095 | 0.0093 | 0.0085 | 0.0097 | 0.0098 | 0.0111 | 0.0054 |
| S Duration in I | 0.0095 | 0.0095 | 0.0111 | 0.0097 | 0.0249 | 0.0096 | 0.0094 | 0.0095 | 0.0094 | 0.0088 | 0.0111 | 0.0049 |
| S Duration in III | 0.0097 | 0.0095 | 0.0090 | 0.0089 | 0.0325 | 0.0093 | 0.0093 | 0.0097 | 0.0092 | 0.0085 | 0.0116 | 0.0074 |
| S Duration in V1 | 0.0087 | 0.0093 | 0.0093 | 0.0057 | 0.0007 | 0.0088 | 0.0092 | 0.0092 | 0.0090 | 0.0098 | 0.0080 | 0.0028 |
| S Duration in V3 | 0.0095 | 0.0090 | 0.0088 | 0.0104 | 0.0005 | 0.0096 | 0.0092 | 0.0094 | 0.0093 | 0.0087 | 0.0084 | 0.0028 |
| S Duration in V4 | 0.0092 | 0.0093 | 0.0090 | 0.0089 | 0.0005 | 0.0092 | 0.0092 | 0.0108 | 0.0090 | 0.0083 | 0.0083 | 0.0028 |
| S Duration in V5 | 0.0094 | 0.0092 | 0.0096 | 0.0104 | 0.0210 | 0.0098 | 0.0092 | 0.0089 | 0.0090 | 0.0093 | 0.0106 | 0.0037 |
| S Duration in V6 | 0.0093 | 0.0093 | 0.0089 | 0.0112 | 0.0054 | 0.0095 | 0.0094 | 0.0093 | 0.0095 | 0.0084 | 0.0090 | 0.0015 |
| S' Duration in V1 | 0.0094 | 0.0084 | 0.0091 | 0.0099 | 0.0009 | 0.0094 | 0.0091 | 0.0092 | 0.0094 | 0.0084 | 0.0083 | 0.0026 |
| S' Duration in V2 | 0.0094 | 0.0093 | 0.0089 | 0.0096 | 0.0010 | 0.0094 | 0.0092 | 0.0092 | 0.0094 | 0.0085 | 0.0084 | 0.0026 |
| QTc Calculation (QTc Bazett) | 0.0103 | 0.0096 | 0.0089 | 0.0133 | 0.0014 | 0.0097 | 0.0092 | 0.0087 | 0.0097 | 0.0096 | 0.0090 | 0.0030 |
| ST at J Point in aVL | 0.0092 | 0.0091 | 0.0090 | 0.0096 | 0.0038 | 0.0062 | 0.0092 | 0.0091 | 0.0093 | 0.0083 | 0.0083 | 0.0018 |
| Minimum ST level in I | 0.0090 | 0.0091 | 0.0090 | 0.0085 | 0.0005 | 0.0094 | 0.0096 | 0.0102 | 0.0082 | 0.0088 | 0.0082 | 0.0028 |
| Minimum ST level in aVR | 0.0095 | 0.0094 | 0.0089 | 0.0086 | 0.0005 | 0.0093 | 0.0094 | 0.0094 | 0.0095 | 0.0087 | 0.0083 | 0.0028 |
| Minimum ST level in aVL | 0.0095 | 0.0084 | 0.0088 | 0.0060 | 0.0408 | 0.0081 | 0.0092 | 0.0067 | 0.0093 | 0.0087 | 0.0116 | 0.0103 |
| Minimum ST level in V2 | 0.0095 | 0.0091 | 0.0089 | 0.0099 | 0.0065 | 0.0094 | 0.0093 | 0.0090 | 0.0093 | 0.0112 | 0.0092 | 0.0012 |
| Minimum ST level in V3 | 0.0090 | 0.0093 | 0.0093 | 0.0035 | 0.0035 | 0.0088 | 0.0093 | 0.0105 | 0.0075 | 0.0076 | 0.0078 | 0.0024 |
| Minimum ST level in V4 | 0.0096 | 0.0094 | 0.0090 | 0.0104 | 0.0005 | 0.0093 | 0.0093 | 0.0084 | 0.0088 | 0.0089 | 0.0084 | 0.0028 |
| Minimum ST level in V5 | 0.0089 | 0.0093 | 0.0092 | 0.0095 | 0.0011 | 0.0094 | 0.0092 | 0.0087 | 0.0096 | 0.0084 | 0.0083 | 0.0026 |
| Maximum ST level in I | 0.0090 | 0.0087 | 0.0090 | 0.0096 | 0.0204 | 0.0089 | 0.0092 | 0.0095 | 0.0093 | 0.0084 | 0.0102 | 0.0036 |
| Maximum ST level in aVR | 0.0088 | 0.0094 | 0.0090 | 0.0100 | 0.0055 | 0.0094 | 0.0090 | 0.0092 | 0.0095 | 0.0082 | 0.0088 | 0.0013 |
| Maximum ST level in aVL | 0.0093 | 0.0092 | 0.0093 | 0.0078 | 0.0036 | 0.0093 | 0.0092 | 0.0085 | 0.0093 | 0.0082 | 0.0084 | 0.0018 |
| Maximum ST level in V1 | 0.0098 | 0.0095 | 0.0092 | 0.0102 | 0.0335 | 0.0095 | 0.0088 | 0.0088 | 0.0094 | 0.0095 | 0.0118 | 0.0076 |
| Maximum ST level in V2 | 0.0093 | 0.0096 | 0.0089 | 0.0087 | 0.0090 | 0.0094 | 0.0092 | 0.0090 | 0.0094 | 0.0080 | 0.0091 | 0.0005 |
| Maximum ST level in V3 | 0.0099 | 0.0095 | 0.0090 | 0.0106 | 0.0285 | 0.0090 | 0.0092 | 0.0089 | 0.0091 | 0.0145 | 0.0118 | 0.0061 |
| Maximum ST level in V4 | 0.0095 | 0.0093 | 0.0089 | 0.0098 | 0.0007 | 0.0089 | 0.0093 | 0.0090 | 0.0093 | 0.0083 | 0.0083 | 0.0027 |
| Maximum ST level in V6 | 0.0096 | 0.0091 | 0.0099 | 0.0081 | 0.0091 | 0.0092 | 0.0088 | 0.0072 | 0.0095 | 0.0078 | 0.0088 | 0.0009 |
| T axis | 0.0091 | 0.0093 | 0.0091 | 0.0055 | 0.0005 | 0.0094 | 0.0092 | 0.0104 | 0.0092 | 0.0087 | 0.0080 | 0.0029 |
| T Area in V3 | 0.0071 | 0.0090 | 0.0087 | 0.0034 | 0.0113 | 0.0093 | 0.0089 | 0.0099 | 0.0090 | 0.0075 | 0.0084 | 0.0021 |
| T' Area in II | 0.0095 | 0.0094 | 0.0088 | 0.0095 | 0.0006 | 0.0095 | 0.0092 | 0.0094 | 0.0091 | 0.0277 | 0.0103 | 0.0067 |
| T' Area in V2 | 0.0096 | 0.0094 | 0.0090 | 0.0122 | 0.0007 | 0.0094 | 0.0094 | 0.0092 | 0.0095 | 0.0084 | 0.0087 | 0.0030 |
| T Area (Full) in V2 | 0.0084 | 0.0089 | 0.0089 | 0.0096 | 0.0046 | 0.0091 | 0.0092 | 0.0095 | 0.0088 | 0.0077 | 0.0085 | 0.0015 |
| T Peak Time in I | 0.0093 | 0.0096 | 0.0090 | 0.0101 | 0.0071 | 0.0095 | 0.0092 | 0.0098 | 0.0094 | 0.0092 | 0.0092 | 0.0008 |
| T Peak Time in aVR | 0.0093 | 0.0094 | 0.0090 | 0.0098 | 0.0005 | 0.0094 | 0.0094 | 0.0091 | 0.0095 | 0.0082 | 0.0084 | 0.0028 |
| T Peak Time in aVL | 0.0097 | 0.0096 | 0.0090 | 0.0101 | 0.0012 | 0.0096 | 0.0090 | 0.0093 | 0.0095 | 0.0087 | 0.0086 | 0.0026 |
| T Peak Time in V1 | 0.0093 | 0.0092 | 0.0089 | 0.0102 | 0.0024 | 0.0092 | 0.0092 | 0.0094 | 0.0093 | 0.0100 | 0.0087 | 0.0023 |
| T Peak Time in V2 | 0.0093 | 0.0096 | 0.0091 | 0.0085 | 0.0005 | 0.0093 | 0.0093 | 0.0091 | 0.0095 | 0.0098 | 0.0084 | 0.0028 |
| T Peak Time in V3 | 0.0095 | 0.0091 | 0.0096 | 0.0097 | 0.0085 | 0.0093 | 0.0091 | 0.0097 | 0.0091 | 0.0094 | 0.0093 | 0.0004 |
| T Peak Amplitude in I | 0.0095 | 0.0093 | 0.0085 | 0.0129 | 0.0006 | 0.0071 | 0.0106 | 0.0087 | 0.0066 | 0.0064 | 0.0080 | 0.0033 |
| T Peak Amplitude in II | 0.0092 | 0.0080 | 0.0091 | 0.0135 | 0.1294 | 0.0091 | 0.0097 | 0.0082 | 0.0074 | 0.0410 | 0.0245 | 0.0382 |
| T Peak Amplitude in aVR | 0.0000 | 0.0073 | 0.0089 | 0.0002 | 0.1001 | 0.0042 | 0.0040 | 0.0074 | 0.0069 | 0.0075 | 0.0147 | 0.0302 |
| T Peak Amplitude in aVL | 0.0091 | 0.0089 | 0.0089 | 0.0116 | 0.0379 | 0.0088 | 0.0090 | 0.0100 | 0.0094 | 0.0083 | 0.0122 | 0.0091 |
| T Peak Amplitude in V6 | 0.0090 | 0.0093 | 0.0090 | 0.0079 | 0.0005 | 0.0094 | 0.0095 | 0.0099 | 0.0093 | 0.0084 | 0.0082 | 0.0028 |
| T' Peak Amplitude in I | 0.0096 | 0.0093 | 0.0089 | 0.0087 | 0.0006 | 0.0094 | 0.0092 | 0.0093 | 0.0093 | 0.0084 | 0.0083 | 0.0027 |
| T' Peak Amplitude in aVL | 0.0094 | 0.0093 | 0.0089 | 0.0096 | 0.0429 | 0.0095 | 0.0092 | 0.0093 | 0.0094 | 0.0086 | 0.0126 | 0.0106 |
| T' Peak Amplitude in V4 | 0.0095 | 0.0095 | 0.0091 | 0.0095 | 0.0017 | 0.0094 | 0.0094 | 0.0093 | 0.0094 | 0.0105 | 0.0087 | 0.0025 |
| T Duration in aVL | 0.0096 | 0.0093 | 0.0089 | 0.0095 | 0.0005 | 0.0096 | 0.0096 | 0.0089 | 0.0094 | 0.0090 | 0.0084 | 0.0028 |
| T Duration in V4 | 0.0078 | 0.0083 | 0.0088 | 0.0071 | 0.0053 | 0.0090 | 0.0091 | 0.0087 | 0.0088 | 0.0095 | 0.0082 | 0.0012 |
| T' Duration in I | 0.0091 | 0.0093 | 0.0089 | 0.0095 | 0.0005 | 0.0094 | 0.0092 | 0.0092 | 0.0094 | 0.0084 | 0.0083 | 0.0028 |
| T' Duration in II | 0.0086 | 0.0093 | 0.0086 | 0.0095 | 0.0027 | 0.0094 | 0.0087 | 0.0084 | 0.0095 | 0.0085 | 0.0083 | 0.0020 |
| T' Duration in V1 | 0.0094 | 0.0093 | 0.0089 | 0.0095 | 0.0005 | 0.0094 | 0.0093 | 0.0108 | 0.0095 | 0.0076 | 0.0084 | 0.0029 |
| T' Duration in V3 | 0.0094 | 0.0093 | 0.0089 | 0.0081 | 0.0008 | 0.0095 | 0.0092 | 0.0092 | 0.0094 | 0.0107 | 0.0085 | 0.0028 |
| T' Duration in V4 | 0.0095 | 0.0092 | 0.0096 | 0.0095 | 0.0037 | 0.0094 | 0.0092 | 0.0092 | 0.0092 | 0.0089 | 0.0087 | 0.0018 |
| T' Duration in V5 | 0.0094 | 0.0093 | 0.0089 | 0.0095 | 0.0150 | 0.0094 | 0.0091 | 0.0100 | 0.0094 | 0.0072 | 0.0097 | 0.0020 |

**Additional file 1: Table S3. The permutation importance of the 109 ECG parameters for cardiovascular death**

|  | Model 1 | Model 2 | Model 3 | Model 4 | Model 5 | Model 6 | Model 7 | Model 8 | Model 9 | Model 10 | Mean | SD |
| --- | --- | --- | --- | --- | --- | --- | --- | --- | --- | --- | --- | --- |
| P-R Interval | 0.0129 | 0.0141 | 0.0141 | 0.0141 | 0.0139 | 0.0138 | 0.0144 | 0.0143 | 0.0144 | 0.0134 | 0.0139 | 0.0005 |
| P' Area in II | 0.0130 | 0.0140 | 0.0136 | 0.0139 | 0.0138 | 0.0139 | 0.0143 | 0.0144 | 0.0143 | 0.0134 | 0.0139 | 0.0004 |
| P' Area in aVF | 0.0132 | 0.0140 | 0.0137 | 0.0139 | 0.0138 | 0.0139 | 0.0143 | 0.0144 | 0.0143 | 0.0135 | 0.0139 | 0.0004 |
| P' Area in V1 | 0.0157 | 0.0139 | 0.0132 | 0.0144 | 0.0135 | 0.0138 | 0.0143 | 0.0142 | 0.0140 | 0.0124 | 0.0139 | 0.0009 |
| P Area (Full) in V1 | 0.0130 | 0.0141 | 0.0144 | 0.0141 | 0.0141 | 0.0150 | 0.0143 | 0.0143 | 0.0144 | 0.0175 | 0.0145 | 0.0012 |
| P' Peak Amplitude in I | 0.0130 | 0.0139 | 0.0136 | 0.0139 | 0.0138 | 0.0139 | 0.0141 | 0.0144 | 0.0143 | 0.0135 | 0.0138 | 0.0004 |
| P' Peak Amplitude in II | 0.0130 | 0.0142 | 0.0136 | 0.0139 | 0.0138 | 0.0139 | 0.0143 | 0.0144 | 0.0143 | 0.0134 | 0.0139 | 0.0004 |
| P' Peak Amplitude in aVF | 0.0135 | 0.0139 | 0.0136 | 0.0139 | 0.0138 | 0.0139 | 0.0143 | 0.0143 | 0.0143 | 0.0137 | 0.0139 | 0.0003 |
| P Duration in V1 | 0.0136 | 0.0146 | 0.0148 | 0.0141 | 0.0138 | 0.0146 | 0.0143 | 0.0144 | 0.0144 | 0.0133 | 0.0142 | 0.0005 |
| P' Duration in I | 0.0131 | 0.0139 | 0.0136 | 0.0139 | 0.0138 | 0.0139 | 0.0143 | 0.0144 | 0.0143 | 0.0134 | 0.0139 | 0.0004 |
| P' Duration in II | 0.0130 | 0.0139 | 0.0136 | 0.0139 | 0.0137 | 0.0139 | 0.0143 | 0.0144 | 0.0144 | 0.0135 | 0.0139 | 0.0004 |
| P' Duration in V1 | 0.0128 | 0.0139 | 0.0140 | 0.0141 | 0.0139 | 0.0140 | 0.0142 | 0.0144 | 0.0144 | 0.0186 | 0.0144 | 0.0015 |
| QRS Area in V2 | 0.0127 | 0.0142 | 0.0150 | 0.0143 | 0.0153 | 0.0160 | 0.0143 | 0.0138 | 0.0140 | 0.0153 | 0.0145 | 0.0009 |
| QRS Area in V3 | 0.0157 | 0.0140 | 0.0149 | 0.0134 | 0.0143 | 0.0139 | 0.0143 | 0.0142 | 0.0140 | 0.0151 | 0.0144 | 0.0007 |
| Q Area in V1 | 0.0130 | 0.0140 | 0.0146 | 0.0139 | 0.0138 | 0.0138 | 0.0144 | 0.0143 | 0.0142 | 0.0135 | 0.0140 | 0.0005 |
| Q Peak Amplitude in V2 | 0.0130 | 0.0146 | 0.0132 | 0.0140 | 0.0138 | 0.0139 | 0.0143 | 0.0142 | 0.0143 | 0.0133 | 0.0139 | 0.0005 |
| Q Duration in V1 | 0.0136 | 0.0153 | 0.0133 | 0.0209 | 0.0216 | 0.0133 | 0.0139 | 0.0133 | 0.0136 | 0.0204 | 0.0159 | 0.0035 |
| Q Duration in V2 | 0.0131 | 0.0150 | 0.0252 | 0.0139 | 0.0138 | 0.0140 | 0.0143 | 0.0140 | 0.0137 | 0.0143 | 0.0151 | 0.0036 |
| R Area in V1 | 0.0128 | 0.0142 | 0.0134 | 0.0138 | 0.0136 | 0.0140 | 0.0143 | 0.0141 | 0.0143 | 0.0132 | 0.0138 | 0.0005 |
| R Area in V5 | 0.0181 | 0.0149 | 0.0140 | 0.0129 | 0.0139 | 0.0140 | 0.0143 | 0.0142 | 0.0142 | 0.0171 | 0.0148 | 0.0016 |
| R Area in V6 | 0.0167 | 0.0140 | 0.0138 | 0.0199 | 0.0157 | 0.0162 | 0.0143 | 0.0144 | 0.0143 | 0.0138 | 0.0153 | 0.0019 |
| R Peak Time in V3 | 0.0137 | 0.0139 | 0.0136 | 0.0141 | 0.0145 | 0.0138 | 0.0143 | 0.0144 | 0.0143 | 0.0136 | 0.0140 | 0.0003 |
| Max R Amplitude in V1 | 0.0146 | 0.0145 | 0.0190 | 0.0162 | 0.0160 | 0.0158 | 0.0142 | 0.0143 | 0.0145 | 0.0194 | 0.0159 | 0.0019 |
| R Duration in V3 | 0.0128 | 0.0142 | 0.0138 | 0.0136 | 0.0140 | 0.0139 | 0.0143 | 0.0144 | 0.0143 | 0.0141 | 0.0139 | 0.0005 |
| R' Duration in V6 | 0.0133 | 0.0140 | 0.0135 | 0.0144 | 0.0138 | 0.0139 | 0.0143 | 0.0144 | 0.0143 | 0.0133 | 0.0139 | 0.0004 |
| S Area in V1 | 0.0194 | 0.0165 | 0.0160 | 0.0151 | 0.0150 | 0.0174 | 0.0143 | 0.0142 | 0.0145 | 0.0223 | 0.0165 | 0.0026 |
| S Area in V3 | 0.0129 | 0.0140 | 0.0136 | 0.0142 | 0.0146 | 0.0140 | 0.0143 | 0.0143 | 0.0143 | 0.0140 | 0.0140 | 0.0005 |
| S Area in V4 | 0.0152 | 0.0136 | 0.0168 | 0.0140 | 0.0139 | 0.0152 | 0.0143 | 0.0141 | 0.0141 | 0.0142 | 0.0145 | 0.0010 |
| S' Area in aVL | 0.0130 | 0.0139 | 0.0136 | 0.0141 | 0.0137 | 0.0139 | 0.0143 | 0.0143 | 0.0143 | 0.0134 | 0.0139 | 0.0004 |
| S' Area in V1 | 0.0128 | 0.0139 | 0.0136 | 0.0139 | 0.0138 | 0.0142 | 0.0143 | 0.0144 | 0.0143 | 0.0134 | 0.0139 | 0.0005 |
| Max S Amplitude in V4 | 0.0138 | 0.0151 | 0.0136 | 0.0139 | 0.0141 | 0.0140 | 0.0143 | 0.0144 | 0.0143 | 0.0136 | 0.0141 | 0.0004 |
| S Duration in V3 | 0.0147 | 0.0139 | 0.0142 | 0.0138 | 0.0137 | 0.0140 | 0.0143 | 0.0142 | 0.0143 | 0.0140 | 0.0141 | 0.0003 |
| S Duration in V4 | 0.0137 | 0.0151 | 0.0142 | 0.0142 | 0.0141 | 0.0157 | 0.0140 | 0.0142 | 0.0143 | 0.0147 | 0.0144 | 0.0006 |
| S' Duration in V1 | 0.0131 | 0.0139 | 0.0139 | 0.0141 | 0.0138 | 0.0144 | 0.0143 | 0.0143 | 0.0143 | 0.0139 | 0.0140 | 0.0004 |
| QTc Calculation (QTc Bazett) | 0.0128 | 0.0148 | 0.0140 | 0.0140 | 0.0161 | 0.0138 | 0.0143 | 0.0144 | 0.0144 | 0.0129 | 0.0142 | 0.0009 |
| ST at J Point in V3 | 0.0129 | 0.0139 | 0.0136 | 0.0140 | 0.0142 | 0.0139 | 0.0143 | 0.0143 | 0.0144 | 0.0135 | 0.0139 | 0.0005 |
| ST at J Point in V4 | 0.0131 | 0.0139 | 0.0137 | 0.0141 | 0.0139 | 0.0139 | 0.0143 | 0.0144 | 0.0143 | 0.0134 | 0.0139 | 0.0004 |
| Minimum ST level in I | 0.0134 | 0.0139 | 0.0136 | 0.0139 | 0.0138 | 0.0138 | 0.0143 | 0.0144 | 0.0144 | 0.0137 | 0.0139 | 0.0003 |
| Minimum ST level in aVR | 0.0130 | 0.0140 | 0.0137 | 0.0140 | 0.0138 | 0.0138 | 0.0143 | 0.0143 | 0.0143 | 0.0133 | 0.0139 | 0.0004 |
| Minimum ST level in V5 | 0.0128 | 0.0139 | 0.0135 | 0.0140 | 0.0137 | 0.0138 | 0.0143 | 0.0143 | 0.0143 | 0.0135 | 0.0138 | 0.0005 |
| Minimum ST level in V6 | 0.0128 | 0.0140 | 0.0141 | 0.0142 | 0.0137 | 0.0139 | 0.0143 | 0.0143 | 0.0143 | 0.0141 | 0.0140 | 0.0005 |
| Maximum ST level in I | 0.0591 | 0.0153 | 0.0206 | 0.0157 | 0.0210 | 0.0145 | 0.0139 | 0.0140 | 0.0125 | 0.0132 | 0.0200 | 0.0141 |
| Maximum ST level in II | 0.0164 | 0.0139 | 0.0169 | 0.0141 | 0.0150 | 0.0141 | 0.0143 | 0.0139 | 0.0144 | 0.0205 | 0.0154 | 0.0021 |
| Maximum ST level in aVR | 0.0133 | 0.0140 | 0.0137 | 0.0141 | 0.0138 | 0.0139 | 0.0143 | 0.0144 | 0.0144 | 0.0136 | 0.0140 | 0.0004 |
| Maximum ST level in aVL | 0.0128 | 0.0141 | 0.0135 | 0.0140 | 0.0138 | 0.0141 | 0.0143 | 0.0144 | 0.0143 | 0.0136 | 0.0139 | 0.0005 |
| Maximum ST level in V3 | 0.0149 | 0.0197 | 0.0133 | 0.0142 | 0.0141 | 0.0147 | 0.0141 | 0.0143 | 0.0143 | 0.0137 | 0.0147 | 0.0018 |
| T axis | 0.0149 | 0.0142 | 0.0139 | 0.0140 | 0.0151 | 0.0141 | 0.0143 | 0.0142 | 0.0144 | 0.0148 | 0.0144 | 0.0004 |
| T' Area in V1 | 0.0129 | 0.0139 | 0.0136 | 0.0144 | 0.0138 | 0.0166 | 0.0143 | 0.0143 | 0.0143 | 0.0140 | 0.0142 | 0.0010 |
| T' Area in V2 | 0.0129 | 0.0139 | 0.0136 | 0.0139 | 0.0138 | 0.0158 | 0.0143 | 0.0143 | 0.0153 | 0.0133 | 0.0141 | 0.0009 |
| T Peak Time in I | 0.0135 | 0.0139 | 0.0138 | 0.0141 | 0.0138 | 0.0152 | 0.0142 | 0.0142 | 0.0143 | 0.0160 | 0.0143 | 0.0007 |
| T Peak Time in II | 0.0128 | 0.0144 | 0.0137 | 0.0140 | 0.0138 | 0.0139 | 0.0143 | 0.0144 | 0.0143 | 0.0136 | 0.0139 | 0.0005 |
| T Peak Time in III | 0.0138 | 0.0144 | 0.0137 | 0.0141 | 0.0138 | 0.0139 | 0.0143 | 0.0143 | 0.0144 | 0.0136 | 0.0140 | 0.0003 |
| T Peak Time in aVR | 0.0128 | 0.0139 | 0.0135 | 0.0140 | 0.0137 | 0.0138 | 0.0143 | 0.0144 | 0.0143 | 0.0133 | 0.0138 | 0.0005 |
| T Peak Time in aVL | 0.0130 | 0.0139 | 0.0137 | 0.0139 | 0.0139 | 0.0138 | 0.0143 | 0.0145 | 0.0143 | 0.0137 | 0.0139 | 0.0004 |
| T Peak Time in V2 | 0.0128 | 0.0140 | 0.0137 | 0.0139 | 0.0146 | 0.0139 | 0.0142 | 0.0144 | 0.0143 | 0.0145 | 0.0140 | 0.0005 |
| T Peak Time in V3 | 0.0128 | 0.0145 | 0.0142 | 0.0139 | 0.0139 | 0.0145 | 0.0143 | 0.0143 | 0.0143 | 0.0144 | 0.0141 | 0.0005 |
| T Peak Time in V5 | 0.0133 | 0.0139 | 0.0137 | 0.0140 | 0.0137 | 0.0139 | 0.0143 | 0.0145 | 0.0143 | 0.0133 | 0.0139 | 0.0004 |
| T Peak Time in V6 | 0.0129 | 0.0155 | 0.0150 | 0.0139 | 0.0140 | 0.0148 | 0.0143 | 0.0144 | 0.0144 | 0.0135 | 0.0143 | 0.0007 |
| T Peak Amplitude in I | 0.0130 | 0.0140 | 0.0143 | 0.0140 | 0.0137 | 0.0142 | 0.0143 | 0.0143 | 0.0145 | 0.0137 | 0.0140 | 0.0004 |
| T Peak Amplitude in II | 0.0141 | 0.0142 | 0.0142 | 0.0142 | 0.0138 | 0.0139 | 0.0143 | 0.0144 | 0.0140 | 0.0136 | 0.0141 | 0.0002 |
| T Peak Amplitude in aVR | 0.0128 | 0.0138 | 0.0134 | 0.0140 | 0.0139 | 0.0138 | 0.0143 | 0.0143 | 0.0145 | 0.0132 | 0.0138 | 0.0005 |
| T Peak Amplitude in aVL | 0.0147 | 0.0146 | 0.0142 | 0.0170 | 0.0167 | 0.0164 | 0.0143 | 0.0140 | 0.0143 | 0.0131 | 0.0149 | 0.0013 |
| T Peak Amplitude in V6 | 0.0128 | 0.0142 | 0.0148 | 0.0141 | 0.0138 | 0.0145 | 0.0143 | 0.0144 | 0.0143 | 0.0137 | 0.0141 | 0.0006 |
| T' Peak Amplitude in I | 0.0130 | 0.0139 | 0.0137 | 0.0139 | 0.0138 | 0.0139 | 0.0143 | 0.0143 | 0.0143 | 0.0134 | 0.0139 | 0.0004 |
| T' Peak Amplitude in II | 0.0133 | 0.0139 | 0.0137 | 0.0139 | 0.0138 | 0.0139 | 0.0143 | 0.0144 | 0.0143 | 0.0134 | 0.0139 | 0.0004 |
| T' Peak Amplitude in V6 | 0.0130 | 0.0140 | 0.0136 | 0.0140 | 0.0140 | 0.0139 | 0.0143 | 0.0144 | 0.0143 | 0.0134 | 0.0139 | 0.0004 |
| T Duration in V4 | 0.0167 | 0.0139 | 0.0139 | 0.0139 | 0.0138 | 0.0141 | 0.0143 | 0.0143 | 0.0143 | 0.0149 | 0.0144 | 0.0009 |
| T' Duration in V2 | 0.0131 | 0.0143 | 0.0138 | 0.0139 | 0.0138 | 0.0134 | 0.0143 | 0.0144 | 0.0143 | 0.0140 | 0.0139 | 0.0004 |
| T' Duration in V3 | 0.0130 | 0.0139 | 0.0136 | 0.0139 | 0.0138 | 0.0141 | 0.0143 | 0.0144 | 0.0143 | 0.0135 | 0.0139 | 0.0004 |
| T' Duration in V4 | 0.0128 | 0.0139 | 0.0137 | 0.0140 | 0.0138 | 0.0138 | 0.0143 | 0.0144 | 0.0143 | 0.0139 | 0.0139 | 0.0005 |
| Minimum ST level in V2 | 0.0029 | 0.0085 | 0.0098 | 0.0092 | 0.0091 | 0.0092 | 0.0091 | 0.0092 | 0.0091 | 0.0092 | 0.0085 | 0.0020 |
| Minimum ST level in V3 | 0.0028 | 0.0086 | 0.0092 | 0.0091 | 0.0088 | 0.0092 | 0.0090 | 0.0092 | 0.0091 | 0.0091 | 0.0084 | 0.0020 |
| Minimum ST level in V4 | 0.0026 | 0.0083 | 0.0091 | 0.0092 | 0.0088 | 0.0092 | 0.0089 | 0.0092 | 0.0091 | 0.0091 | 0.0084 | 0.0020 |
| Minimum ST level in V5 | 0.0035 | 0.0083 | 0.0092 | 0.0093 | 0.0094 | 0.0092 | 0.0090 | 0.0093 | 0.0092 | 0.0091 | 0.0086 | 0.0018 |
| Maximum ST level in I | 0.0026 | 0.0082 | 0.0088 | 0.0093 | 0.0074 | 0.0089 | 0.0088 | 0.0083 | 0.0091 | 0.0091 | 0.0081 | 0.0020 |
| Maximum ST level in aVR | 0.0027 | 0.0083 | 0.0093 | 0.0093 | 0.0111 | 0.0092 | 0.0091 | 0.0093 | 0.0092 | 0.0091 | 0.0087 | 0.0022 |
| Maximum ST level in aVL | 0.0025 | 0.0085 | 0.0091 | 0.0092 | 0.0090 | 0.0092 | 0.0089 | 0.0090 | 0.0091 | 0.0091 | 0.0084 | 0.0021 |
| Maximum ST level in V1 | 0.0073 | 0.0091 | 0.0093 | 0.0090 | 0.0085 | 0.0092 | 0.0090 | 0.0092 | 0.0092 | 0.0091 | 0.0089 | 0.0006 |
| Maximum ST level in V2 | 0.0114 | 0.0080 | 0.0083 | 0.0092 | 0.0087 | 0.0093 | 0.0089 | 0.0091 | 0.0091 | 0.0091 | 0.0091 | 0.0009 |
| Maximum ST level in V3 | 0.0107 | 0.0088 | 0.0086 | 0.0092 | 0.0088 | 0.0093 | 0.0090 | 0.0092 | 0.0091 | 0.0092 | 0.0092 | 0.0006 |
| Maximum ST level in V4 | 0.0040 | 0.0085 | 0.0093 | 0.0092 | 0.0085 | 0.0091 | 0.0089 | 0.0092 | 0.0091 | 0.0092 | 0.0085 | 0.0016 |
| Maximum ST level in V6 | 0.0031 | 0.0083 | 0.0091 | 0.0092 | 0.0098 | 0.0092 | 0.0092 | 0.0093 | 0.0092 | 0.0092 | 0.0086 | 0.0020 |
| T axis | 0.0024 | 0.0081 | 0.0095 | 0.0092 | 0.0096 | 0.0091 | 0.0088 | 0.0092 | 0.0090 | 0.0091 | 0.0084 | 0.0021 |
| T Area in V3 | 0.0027 | 0.0092 | 0.0092 | 0.0092 | 0.0097 | 0.0092 | 0.0091 | 0.0093 | 0.0091 | 0.0091 | 0.0086 | 0.0021 |
| T' Area in II | 0.0028 | 0.0082 | 0.0091 | 0.0092 | 0.0099 | 0.0097 | 0.0090 | 0.0092 | 0.0091 | 0.0091 | 0.0085 | 0.0021 |
| T' Area in V2 | 0.0028 | 0.0084 | 0.0093 | 0.0092 | 0.0090 | 0.0098 | 0.0089 | 0.0093 | 0.0110 | 0.0092 | 0.0087 | 0.0022 |
| T Area (Full) in V2 | 0.0030 | 0.0104 | 0.0093 | 0.0087 | 0.0109 | 0.0091 | 0.0095 | 0.0091 | 0.0090 | 0.0092 | 0.0088 | 0.0022 |
| T Peak Time in I | 0.0025 | 0.0082 | 0.0066 | 0.0091 | 0.0085 | 0.0096 | 0.0091 | 0.0092 | 0.0092 | 0.0092 | 0.0081 | 0.0022 |
| T Peak Time in aVR | 0.0025 | 0.0082 | 0.0080 | 0.0091 | 0.0085 | 0.0101 | 0.0094 | 0.0092 | 0.0092 | 0.0092 | 0.0083 | 0.0021 |
| T Peak Time in aVL | 0.0022 | 0.0082 | 0.0092 | 0.0092 | 0.0088 | 0.0093 | 0.0090 | 0.0091 | 0.0092 | 0.0092 | 0.0083 | 0.0022 |
| T Peak Time in V1 | 0.0025 | 0.0111 | 0.0091 | 0.0093 | 0.0090 | 0.0091 | 0.0090 | 0.0093 | 0.0091 | 0.0094 | 0.0087 | 0.0023 |
| T Peak Time in V2 | 0.0025 | 0.0082 | 0.0082 | 0.0091 | 0.0084 | 0.0098 | 0.0092 | 0.0093 | 0.0091 | 0.0091 | 0.0083 | 0.0021 |
| T Peak Time in V3 | 0.0028 | 0.0087 | 0.0094 | 0.0091 | 0.0093 | 0.0095 | 0.0093 | 0.0092 | 0.0091 | 0.0091 | 0.0086 | 0.0020 |
| T Peak Amplitude in I | 0.0053 | 0.0089 | 0.0093 | 0.0092 | 0.0106 | 0.0093 | 0.0093 | 0.0093 | 0.0091 | 0.0091 | 0.0089 | 0.0014 |
| T Peak Amplitude in II | 0.0026 | 0.0081 | 0.0101 | 0.0092 | 0.0102 | 0.0095 | 0.0090 | 0.0092 | 0.0091 | 0.0089 | 0.0086 | 0.0022 |
| T Peak Amplitude in aVR | 0.0032 | 0.0091 | 0.0086 | 0.0092 | 0.0083 | 0.0092 | 0.0089 | 0.0092 | 0.0091 | 0.0092 | 0.0084 | 0.0019 |
| T Peak Amplitude in aVL | 0.0050 | 0.0130 | 0.0079 | 0.0088 | 0.0106 | 0.0093 | 0.0102 | 0.0089 | 0.0091 | 0.0087 | 0.0092 | 0.0020 |
| T Peak Amplitude in V6 | 0.0025 | 0.0083 | 0.0092 | 0.0092 | 0.0093 | 0.0092 | 0.0090 | 0.0092 | 0.0091 | 0.0092 | 0.0084 | 0.0021 |
| T' Peak Amplitude in I | 0.0028 | 0.0082 | 0.0092 | 0.0092 | 0.0096 | 0.0092 | 0.0089 | 0.0092 | 0.0091 | 0.0092 | 0.0085 | 0.0020 |
| T' Peak Amplitude in aVL | 0.0027 | 0.0082 | 0.0091 | 0.0092 | 0.0088 | 0.0092 | 0.0090 | 0.0093 | 0.0091 | 0.0092 | 0.0084 | 0.0020 |
| T' Peak Amplitude in V4 | 0.0027 | 0.0082 | 0.0091 | 0.0092 | 0.0089 | 0.0091 | 0.0090 | 0.0091 | 0.0091 | 0.0092 | 0.0084 | 0.0020 |
| T Duration in aVL | 0.0085 | 0.0083 | 0.0094 | 0.0092 | 0.0091 | 0.0092 | 0.0090 | 0.0093 | 0.0089 | 0.0091 | 0.0090 | 0.0003 |
| T Duration in V4 | 0.0028 | 0.0083 | 0.0090 | 0.0094 | 0.0133 | 0.0088 | 0.0089 | 0.0092 | 0.0092 | 0.0095 | 0.0088 | 0.0025 |
| T' Duration in I | 0.0028 | 0.0082 | 0.0093 | 0.0092 | 0.0088 | 0.0092 | 0.0089 | 0.0092 | 0.0091 | 0.0091 | 0.0084 | 0.0020 |
| T' Duration in II | 0.0028 | 0.0083 | 0.0091 | 0.0092 | 0.0085 | 0.0094 | 0.0090 | 0.0092 | 0.0091 | 0.0091 | 0.0084 | 0.0020 |
| T' Duration in V1 | 0.0027 | 0.0084 | 0.0092 | 0.0092 | 0.0088 | 0.0092 | 0.0088 | 0.0092 | 0.0091 | 0.0092 | 0.0084 | 0.0020 |
| T' Duration in V3 | 0.0029 | 0.0083 | 0.0092 | 0.0092 | 0.0092 | 0.0099 | 0.0091 | 0.0093 | 0.0093 | 0.0092 | 0.0086 | 0.0020 |
| T' Duration in V4 | 0.0026 | 0.0082 | 0.0091 | 0.0092 | 0.0089 | 0.0092 | 0.0089 | 0.0093 | 0.0091 | 0.0092 | 0.0084 | 0.0021 |
| T' Duration in V5 | 0.0028 | 0.0083 | 0.0091 | 0.0092 | 0.0075 | 0.0092 | 0.0090 | 0.0092 | 0.0089 | 0.0091 | 0.0082 | 0.0020 |

**Additional file 1: Table S4. The c-statistics of the predictive models for all-cause and cardiovascular death by support vector machine**

|  | All-cause death | | Cardiovascular death | |
| --- | --- | --- | --- | --- |
| Model | Training model | Testing model | Training model | Testing model |
| 1 | 0.916 | 0.999 | 0.880 | 0.864 |
| 2 | 0.875 | 0.850 | 0.855 | 0.828 |
| 3 | 0.894 | 0.967 | 0.888 | 0.876 |
| 4 | 0.848 | 0.994 | 0.878 | 0.932 |
| 5 | 0.827 | 0.995 | 0.884 | 0.938 |
| 6 | 0.888 | 0.668 | 0.849 | 0.999 |
| 7 | 0.873 | 0.959 | 0.808 | 0.825 |
| 8 | 0.884 | 0.938 | 0.898 | 0.801 |
| 9 | 0.898 | 0.946 | 0.827 | 0.988 |
| 10 | 0.905 | 0.953 | 0.849 | 0.923 |
| Mean | 0.881 | 0.927 | 0.862 | 0.897 |
| SD | 0.027 | 0.101 | 0.029 | 0.069 |
